# Vascular calcification: from the perspective of crosstalk

**DOI:** 10.1186/s43556-023-00146-y

**Published:** 2023-10-18

**Authors:** Shiqi Yang, Zhaolin Zeng, Qing Yuan, Qian Chen, Zuo Wang, Hui Xie, Jianghua Liu

**Affiliations:** 1 Department of Metabolism and Endocrinology, Hengyang Medical School, The First Affiliated Hospital, University of South China, Hengyang, 421001 Hunan China; 2 Department of Clinical Laboratory Medicine, Hengyang Medical School, The First Affiliated Hospital, University of South China, Hengyang, 421001 Hunan China; 3 Institute of Cardiovascular Disease, Key Lab for Arteriosclerology of Hunan Province, Hengyang Medical School, University of South China, Hengyang, 421001 Hunan China; 4 Department of Orthopaedics, Movement System Injury and Repair Research Centre, Xiangya Hospital, Central South University, Changsha, Hunan Province China

**Keywords:** Vascular calcification, Crosstalk, Microenvironment, Extracellular vesicles

## Abstract

Vascular calcification (VC) is highly correlated with cardiovascular disease morbidity and mortality, but anti-VC treatment remains an area to be tackled due to the ill-defined molecular mechanisms. Regardless of the type of VC, it does not depend on a single cell but involves multi-cells/organs to form a complex cellular communication network through the vascular microenvironment to participate in the occurrence and development of VC. Therefore, focusing only on the direct effect of pathological factors on vascular smooth muscle cells (VSMCs) tends to overlook the combined effect of other cells and VSMCs, including VSMCs-VSMCs, ECs-VMSCs, Macrophages-VSMCs, etc. Extracellular vesicles (EVs) are a collective term for tiny vesicles with a membrane structure that are actively secreted by cells, and almost all cells secrete EVs. EVs docked on the surface of receptor cells can directly mediate signal transduction or transfer their contents into the cell to elicit a functional response from the receptor cells. They have been proven to participate in the VC process and have also shown attractive therapeutic prospects. Based on the advantages of EVs and the ability to be detected in body fluids, they may become a novel therapeutic agent, drug delivery vehicle, diagnostic and prognostic biomarker, and potential therapeutic target in the future. This review focuses on the new insight into VC molecular mechanisms from the perspective of crosstalk, summarizes how multi-cells/organs interactions communicate via EVs to regulate VC and the emerging potential of EVs as therapeutic methods in VC. We also summarize preclinical experiments on crosstalk-based and the current state of clinical studies on VC-related measures.

## Introduction

With the acceleration of the global aging process and the increasing prevalence of metabolic diseases, the prevalence of vascular calcification (VC) is increasing dramatically [1, 2]. VC is common in patients with atherosclerosis, diabetes, chronic kidney disease (CKD), and other advanced diseases [3]. VC can reduce the elasticity and compliance of the vascular wall, increase the instability of atherosclerotic plaque, aggravate cardiac afterload, and finally lead to intravascular thrombosis, vascular occlusion, left ventricular hypertrophy, heart failure, and acute myocardial infarction [4]. It is an independent predictor of cardiovascular morbidity and mortality and a harbinger of future coronary heart disease, stroke, lower limb amputations, and other diseases [4, 5].

Initially, VC was thought to be a passive process due to the pathological mineral deposition in the vascular system. VC is now widely recognized as an active, dynamic, and complex pathological process regulated by numerous factors, with VSMCs playing a key driving role [6]. In addition to VSMCs, endothelial cells, pericytes, macrophages, bone marrow-derived mesenchymal stem cells, and so on, can also be involved in VC [7–10]. Current understanding of the pathogenesis of VC includes calcium and phosphorus imbalance [11–13], VSMCs transdifferentiation [14, 15], extracellular matrix [16], EVs [17], bone homeostasis imbalance [18–22], inflammation [23], epigenetics (DNA methylation and demethylation, histone modification, non-coding RNA) [24–29], autophagy [30], oxidative stress [31], mitochondrial dysfunction [24], iron death [32] and pyroptosis [33], etc. Despite some progress in very recent years, VC remains difficult to operate and has poor outcomes and prognoses because the exact mechanisms of VC remain unclear [34].

Crosstalk refers to information communication between cells, the interaction between information substances, and the interaction between information transduction pathways or channels. Therefore, crosstalk is a multi-level and time–space characterization of the network involving information communication, coordination and interaction. It has been demonstrated that the occurrence of any type of VC does not occur in a single cell/organ, but involves multi-cells/organs and systems [35]. Crosstalk enables cell-to-cell/organ-to-organ interactions in VC through a variety of biomolecules and signaling pathways. For example, endothelial cells can interact with VSMCs through myoendothelial gap junction (MEGJ), release various biomolecules and EVs under pathological stimulation, and then affect the occurrence and development of VC [36].

In recent years, EVs have been found to play a crucial role in cross-border crosstalk. There is compelling evidence that EVs play an important regulatory role not only in diseases such as bone diseases, tumours and metabolic diseases [37–39], but also in cardiovascular diseases (CVD). EVs are secreted from cells into circulation in a regulated manner and are captured by distant cells to exert their biological effects [39, 40]. In fact, EVs carry biologically active substances, such as nucleic acids, proteins, lipids, and so on [41–45], which can affect target cells through the following mechanisms:(1) EVs activate target cell downstream signaling pathways through specific binding of surface ligands to target cell receptors;(2) EVs directly transfer the receptor in the activated state to the target cells, thereby activating downstream signaling pathways; and (3) EVs transfer their contents into target cells through direct fusion with the plasma membrane or endocytosis, and mediate target cell biological effects by regulating downstream signaling pathways in target cells [46, 47]. Under different physiopathological conditions, the amount of EVs secreted by maternal cells and their contents are altered and the results of EVs targeting cells such as VSMCs or ECs are different. In this review, we summarize a new perspective of EVs-mediated intercellular/interorgan material communication to elucidate the molecular mechanism of VC and "cross-talk"-based therapeutic strategies.

## Overview of extracellular vesicles

### Discovery and nomenclature of EVs

Extracellular vesicles(EVs) are nanoparticles with lipid bilayer structure and no nucleus; and are a heterogeneous population of membrane-bound vesicles (heterogeneity in size, content, function, and origin) [40]. Exosomes were originally discovered by Dr. Rose Johnstone in order to understand the biological process of reticulocyte transformation into mature red blood cells and thought that they might be one of the ways of cell excretion of waste products, but recent studies have shown that they have multiple functions, contrary to the original view that exosomes are waste excretion [48].

The classification of extracellular vesicles (EVs) is constantly evolving, and in the past different names have been used in the litetature to denote these EVs: according to their size, they are called microparticles, microvesicles, nanovesicles and nanopartices; according to their possible functions, they are called matrix vesicles, argosomes, tolerosomes; as they are secreted outside the cell, they are called etosomes, exosomes, exovesicles, exosome-like vesicles, and so on [42, 49, 50]. In fact, there are no universal markers to distinguish between different types of EVs, and each has overlapping characteristics [51]. Therefore, the biological classification and nomenclature of EVs is complex and controversial [50], and isolating specific EV subtypes is challenging. The International Society for Extracellular Vesicles (ISEV) recommends the use of characteristics such as the size (< 200 nm for small vesicles and > 200 nm for medium/large vesicles) or density and biochemical composition (EVs expressing CD63/CD81 or Annexin A5), among other physicochemical properties, to characterize EVs [51].

### Biogenesis, release and internalization of EVs

According to their mechanisms of occurrence, EVs can be divided into three types: Exosomes (30–150 nm, endocytic pathway of EVs origin), Microvesicles (100–1000 nm EVs sprouting from the plasma membrane), Apoptotic bodies (Abs, 1–5 μm EVs from apoptotic cells) [52–54]. Exosomes originate from endocytosis. Endosomal membranes bud inward to form intraluminal vesicles (ILVs) in the lumen of multivesicular bodies (MVBs) [55]. ILV is released as exosomes by plasma membrane fusion, involving ESCRT (endosomal sorting complex required for transport)-dependent mechanisms, ESCRT-independent mechanisms and other nonclassical pathways [56–58]. The ESCRT-independent exosome synthesis pathway requires ceramide [59–61]. Release of cellular exosomes was significantly reduced after inhibition of neurtral sphingomyelinase (n-SMase) [62], a key enzyme in ceramide production [59–61]. GW4869 is a non-competitive neutral sphingomyelinase inhibitor that prevents ILVs formation capable of blocking exosome production [63–66]. Several studies have shown that calcification can be significantly ameliorated after the use of GW4869 at the cellular level or in mice models [67–69], or that GW4869 partially reverses substances that act as inhibitors of calcification [70, 71]. These two reviews provide a detailed assessment of the major drugs currently interfering with the biosynthesis or release of EVs [65, 72]. By using confocal microscopy it was observed that some MVBs can also be degraded by interacting with lysosomes [73]. Unlike the exosomes, microvesicles are formed by direct germination and division of the plasma membrane [74]. During microvesicle biogenesis, the interaction between the cytoskeleton and the plasma membrane weakens and several proteins such as calpain or lipid transferases are activated [58]. This remodeling of the cytoskeleton is associated with an increase in intracellular calcium concentration, which in turn induces phosphatidylserine externalization, bud formation, and microvesicle secretion [75]. Unlike exosomes and microvesicles, which are secreted during normal cellular processes, apoptotic bodies are only released upon apoptosis [58]. Apoptotic bodies are formed due to cytoskeleton destruction of apoptotic cells, swelling of plasma membrane and separation of plasma membrane [55, 76].

**Fig. 1:**
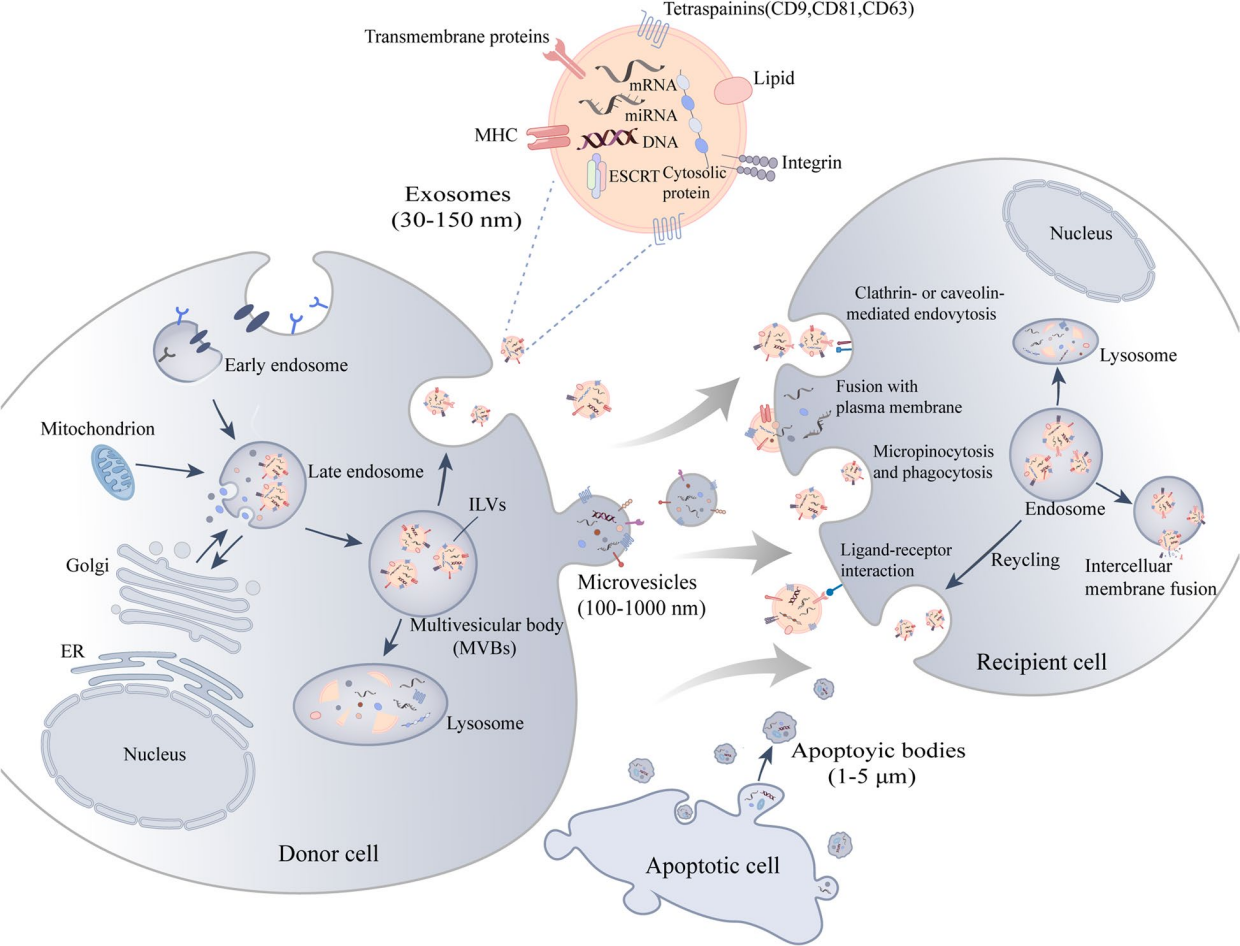
Biogenesis, release and internalization of EVs. EVs can be divided into three types according to their mechanisms: Exosomes (30–150 nm), Microvesicles (100–1000 nm), Apoptotic bodies (Abs, 1–5 μm). Exosomes originate from endocytosis. Endosomal membranes bud inward to form intraluminal vesicles (ILVs) in the lumen of multivesicular bodies (MVBs). ILV is released as exosomes by plasma membrane fusion, involving ESCRT (endosomal sorting complex required for transport)-dependent mechanisms, ESCRT-independent mechanisms and other nonclassical pathways. Some MVBs can also be degraded by interacting with lysosomes. Proteins of the tetraspanin family such as CD9, CD63 and CD81, are abundant in exosomes and are widely recognized as markers of exosomes. Microvesicles are formed by direct germination and division of the plasma membrane. Abs are formed due to the cytoskeleton destruction of apoptotic cells, swelling of the plasma membrane and separation of the plasma membrane. Mechanisms of EVs internalization by target cells: Endocytosis mediated by clathrin or caveolin; fusion with plasma membrane; micropinocytosis and phagocytosis; ligand-receptor interaction. After being taken up by target cells, the contents (such as proteins, mRNAs, non-coding RNAs, and lipids) of EVs are released to regulate the translation, metabolism, growth and development of target cells

CD29 is a marker for both microvesicles and exosomes, while CD29^+^EVs can be used to distinguish between microvesicles and exosomes when combined with other protein markers; for example, Tetraspanins, including CD63, are thought to be mostly present in exosomes but mostly absent in microvesicles [77]. Furthermore, proteins of the tetraspanin family such as CD9 and CD81 are also abundant in exosomes and are also widely considered as markers [78]. The membrane lipid bilayer of EVs also contains integrins, cell adhesion molecules, etc. [78, 79] The first two types of extracellular vesicles are currently being studied, and some researchers refer to these collectively as EVs. Differences in their surface molecules and size may affect the ability to be internalized and studies have shown the mechanisms by which EVs are internalized: ligand-receptor interaction, micropinocytosis, phagocytosis, and endocytosis mediated by target cells via clathrin or caveolin [79–81]; they can also fuse with the cell membrane to release their contents into the cytosol of the target cell [82] (Fig. 1).

EVs come from a wide range of sources as many types of cells can secrete EVs, for example, macrophage, BMSC, VSMC, ECs, VICs, blood cells, etc., and are relatively stable present in a variety of body fluids, including blood, urine, saliva, bile and breast milk, etc. [57]. Numerous studies have shown that EVs can be released in both physiological and pathological settings and act as intercellular communication vehicles to transfer a wide range of biomolecules such as proteins, DNA, mRNAs, non-coding RNAs (ncRNAs: miRNAs, lncRNAs, circRNAs) and lipids from their parent cells to proximal or distal target cells/organs, regulating their activities including translation, metabolism, growth and development, and dynamically reflecting the state of the disease [39, 40]. Therefore, EVs as a medium play an important role in information exchange, which differs from the traditional way of information exchange.

## Overview of the current knowledge of vascular calcification molecular mechanism

### Classification and associated risk factors of VC

**Fig. 2:**
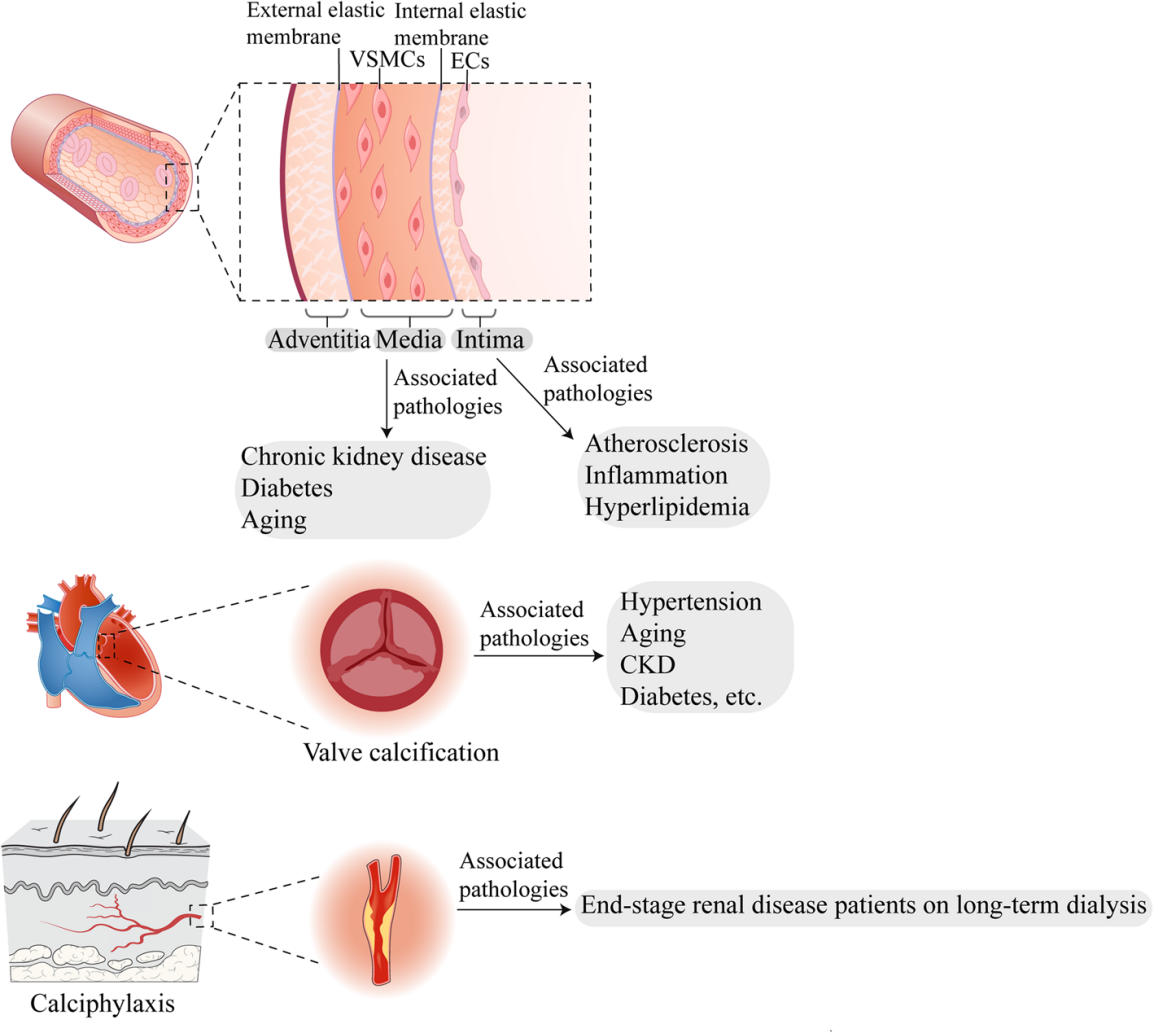
Classification of vascular calcification (VC) and associated risk factors. VC can be divided into five categories according to its site of occurrence and pathophysiological mechanisms: intimal, media, adventitia, valvular calcification (ValvC), and calciphylaxis

The occurrence of vascular calcification is a dynamic process in many aspects, which depends on the change in the microenvironment and the occurrence site. The pathological manifestation is to induce abnormal deposition of calcium, phosphorus, and other minerals in the form of hydroxyapatite on the vascular wall [3]. Abnormal mineralization can occur in all vascular. There are many classification methods for VC according to different causes. VC can be divided into five categories according to its site of occurrence and pathophysiological mechanisms: intimal, media, adventitia, valvular calcification (ValvC), and calciphylaxis [83] (Fig. 2).

Intimal calcification occurs frequently in atherosclerotic lesions and is associated with hyperlipidemia, macrophage infiltration and vascular inflammation [84]. Medial calcification mainly occurs in aging, diabetes and CKD diseases development, and VSMCs play a crucial role in medial calcification [3, 85]. Adventitial calcification is mainly thought to be related to pericytes, but the mechanism of adventitial calcification is still unclear and less studied [35]. Valvular calcification mainly occurs in aortic and mitral valves, and risk factors are often related to hypertension, aging, diabetes, and CKD [86, 87]. Calciphylaxis is a rare vascular calcification syndrome with severe pain, ulcer or necrotic skin injury in multiple parts of the body due to subcutaneous adipose tissue and microvascular occlusion of the dermis [88, 89]. Once calciphylaxis occurs, the disease progresses rapidly and the survival rate is low [88]. This disease has not been accurately recognized at present and usually occurs in patients on long-term dialysis for end-stage renal disease [88].

Calcification also can be divided into physiological calcification and pathological calcification [90]. Physiological calcification occurs in the normal physiological process of bone and tooth [90]. During the initial stages of mineralization of hard tissues such as bone, cartilage and dentin, hard tissue forming cells secrete matrix vesicles to function [91, 92]. For example, bone matrix vesicles are released during bone formation and development by microvilli sprouting at the tip of osteoblasts and participate in the initiation of mineralization by adjusting the ratio of pyrophosphate to phosphate, providing sites for hydroxyapatite crystal nucleation and interacting with extracellular matrix to promote the formation of hydroxyapatite in its cavity [92–94]. This series of processes is known as matrix vesicle-mediated mineralization. Pathological calcification is closely related to diabetes, chronic kidney disease (CKD), aging, advanced atherosclerosis, fatty liver, etc. [3] The mechanisms that promote the initiation and progression of VC are similar to those of physiological bone formation and include osteogenic/chondrogenic transdifferentiation, reduced availability of calcification inhibitors, the release of EVs, and remodeling of the extracellular matrix [95].

### VSMCs-derived MVs mediated mineral deposition

VSMCs are the predominant cell type in the vessel wall, are plastic, and can switch between a contractile and a synthetic phenotype [96]. VSMCs exhibit a contractile phenotype to maintain an anti-VC microenvironment under physiological conditions. Upon abnormal stimulation, VSMCs will switch from a contractile phenotype to a synthetic phenotype (osteogenic/cartilaginous phenotype), which is a necessary condition for VSMC calcification [96, 97]. The phenotypic switch is driven by enhanced expression of osteogenic markers such as alkaline phosphatase (ALP), runt-related transcriptionfactor2 (Runx2), osteocalcin (OCN), bone morphogenetic protein (BMP), osteopontin (OPN), collagen type 1 (COL1) and bone sialoproteins, etc. [40, 98] In addition, there is also suppression of VSMC lineage markers such as sm22α and α-smooth muscle actin (α-SMA), etc. [3, 14, 99] Like Bone cells, VSMCs can also produce matrix EVs-like structures (Matrix vesicles, MVs). Matrix vesicles (30-300 nm) are a specialized type of extracellular vesicles [100]. The analysis of size, shape, lipid and protein content showed that the matrix vesicles were anchored exosomes secreted by cells and that they were homologous structures [101, 102]. Currently, there are no comprehensive definitions and no studies have been done to distinguish MVs from exosomes and microvesicles [101], so they should not be confused. MVs may represent a mixture of exosomes and microvesicles that contain specific components needed to direct ECM mineralization [103, 104].

VSMCs spontaneously release MVs under physiological conditions, and MVs are loaded with mineralization inhibitors (such as endogenous inhibitor MGP, cycle inhibitor Fetuin-A) to prevent mineral nucleation, which is an adaptive response aimed at preventing intracellular calcium overload [105, 106]. Matrix Gla protein (MGP), a vitamin K-dependent protein, is considered a natural calcification inhibitor and is highly expressed in VSMCs and chondrocytes [107]. MGP carboxylated by Vit K2 can not only prevent crystal formation, but also block BMP signal transduction to inhibit VC [107]. Therefore, reducing VC requires maintaining Vit K2 levels and avoiding the use of Vit K antagonists such as warfarin [108].

In contrast, under pathological stimulation (such as high extracellular calcium), VSMCs can secrete mineralized MVs in which MGP expression is absent and the activity of calcification-promoting factor matrix metalloproteinases 2 (MMP2) is increased [109]. When the tunica media calcifies, the degradation of elastin leads to the decrease of elastic fiber cross-linking to promote the deposition of hydroxyapatite crystals, and the elastic degradation products can further promote the transformation of VSMC osteogenic phenotype and aggravate VC [110]. In addition to the loss of calcium-induced inhibitors, VSMC-derived MVs are also involved in key events in the calcification process. Chen et al. [111] suggested that mineralization in VSMC requires both active MVs and the interaction of MVs with COL 1(ECM) to promote the release and aggregation of hydroxyapatite crystals, and the activity of annexin plays an important role in both steps. Previously, Annexin was shown to be a pro-calcification marker for calcifying EVs [109, 112]. AnxA6 and phosphatidylserine forms a protein-lipid complex that nucleates hydroxyapatite, leading to mineral deposition initiating micromineralization [109, 113]. Under the condition of continuous calcium overload, AnxA6 can shuttle to the plasma membrane to regulate calcium homeostasis and vesicle release to maintain intracellular calcium homeostasis and limit cell damage caused by calcium overload [109]. It has been shown that the mineralization of VSMC-MVs is a pathological reaction of intracellular calcium homeostasis disorder. Disturbance of calcium and phosphorus homeostasis is the premise of bone mineralization and vascular calcification development [11, 114]. MVs regulate ion channels that affect the concentration of internal and external calcium and phosphorus to regulate matrix mineralization. In addition, pathological stimulation such as Ca^2+^ and Pi can induce VSMC dedifferentiation and apoptosis, and then VSMC secretes MVs and releases apoptotic bodies into the extracellular matrix, which promotes calcium and phosphorus deposition, thus forming the initial focus of VC and promoting calcification [11, 27].

Gene mutations of proteins in MVs can produce correspond matrix mineralization abnormalities, such as enzyme activity imbalance and ion concentration imbalance [115]. Activation of tissue non-specific alkaline phosphatase (TNAP) in VSMCs precedes vascular calcification in atherosclerosis. TNAP may shift the Pi/PPi balance toward calcification by hydrolyzing the calcification inhibitor inorganic pyrophosphate (PPi) and generating inorganic phosphate (Pi) required for mineralization, which is critical for MVs-mediated hydroxyapatite formation [116]. Anderson et al. [117] demonstrated inadequate bone mineralization in TNAP^-/-^knockout mice and proposed two major hypothesized mechanisms: the first stage is primarily from an inability of initial mineral crystals within MVs to self-nucleate and to proliferate beyond the protective confines of the MVs membrane; the failure of the second stage of mineral formation may be due to the excess of the mineral inhibitor pyrophosphate in the extracellular-fluid around MVs. The activity of TNAP is necessary for both physiological bone mineralization and the induction of pathological calcification. MVs contain another phosphatase, PHOSPHO1, which acts as an alternative supplier of Pi, and plays a role in the initiation of matrix mineralization and may also be involved in MVs-mediated calcification [118–120]. The pathological induction of vascular wall by TNAP and PHOSPHO1 may be an important cause of atherosclerotic calcification [116].

In summary, VSMCs-derived MVs are involved in the initiation of VC. Apoptosis [121, 122], pyroptosis [33], oxidative stress [31, 123], inflammation [23, 124], autophagy [30, 125], microRNAs [27, 126], aging [14, 127], ferroptosis [32, 128], Metabolic reprogramming [129, 130], and epigenetic changes caused by reduced ATP production in mitochondrial insufficiency [24, 25, 131, 132] have also been reported to play an important role in the regulation of VC. The signaling pathways associated with VC such as Msx2, mTOR, MAPK, Wnt, and TGF-β pathways have been extensively elucidated [14, 133].

### Current status and clinical treatment for VC

The current treatment of calcified lesions is mainly mechanical treatment with "coronary artery spinning" and "Intravascular lithotripsy (IVL)" [34, 134–136]. In addition to the treatment of calcification, the risk factors associated with it, such as diabetes, chronic kidney disease, hyperlipidemia and smoking, should be treated and controlled [137–140]. Sodium thiosulphate [141–144], bisphosphonates [145–147], SNF472 [148–150], phosphate binders [151, 152], calcimimetics [153, 154], Denosumab [155] and TNAP inhibitors [156–158] can potentially intervene in VC, but their clinical applications need to be further explored in depth. Prevention and treatment of VC focuses on early monitoring and prevention, correcting disturbances in calcium and phosphorus metabolism, avoiding hypercalcemia, and preventing secondary hyperparathyroidism or hypoparathyroidism. There is a lack of effective therapeutic measures to prevent and treat VC. It has been recognized that the VC process is a complex network, involving multiple cells involved in regulation, such as VSMC to osteogenic phenotype transformation, endothelial dysfunction, EndMT, stem/progenitor cell to osteoblast differentiation, etc. [14, 159, 160]. However, studies only focus on the direct effects of various pathological factors on vascular wall cells (e.g., VSMCs, ECs and macrophages) will tend to ignore the comprehensive effects of multiple cells. Once formed, VC is difficult to reverse, and although there are many studies on how to overcome VC, the complexity and diversity of VC pathophysiology hinders the discovery of optimal drug targets and drug development. Thus, it remains a major clinical challenge, and further insights into the mechanisms of vascular calcification are necessary to potentially trigger the development of multiple types of therapeutic agents. In the past, a large number of studies have focus on the role of EVs in mediating mineral deposition, whereas in recent years it has been found that EVs can mediate cell-to-cell/organ-to-organ crosstalk to propagate calcification. Using the biological characteristics of EVs, the molecular mechanism of VC is elucidated from a new perspective, which may provide a new direction for the treatment and prevention of VC in the future. Improving the understanding of crosstalk between VSMCs and cells/organs will not only help to regulate the risk factors related to the occurrence and development of VC, but also may provide insight into the development of VC treatment.

## The crosstalk in vascular calcification

### Extracellular vesicles mediated crosstalk

In addition to the aggregation of calcified EVs to produce microcalcifications in the ECM as elaborated above, EVs also play an important mediator role in multi-organ/cellular regulation of VC. The vascular wall can be divided into three layers: tunica intima, tunica media and tunica adventitia, and has a variety of functionally active cells [161]. The development of vascular calcification is not dictated by cells in one of the layers of the vessel wall, but rather it tends to function as a whole with cellular crosstalk. Under physiological conditions, vascular cell-derived EVs regulate the homeostasis of the vascular wall, but when subjected to pathological stimuli, such as risk factors associated with cardiovascular diseases: aging, diabetes, CKD, smoking, lipoprotein(a) and inflammation, etc., the biological properties of EVs can be altered to acquire pro-calcification potential and increase their release [17, 46, 162–165], and mediate communication between vascular wall cells through the EVs thereby affecting environmental homeostasis and VSMC phenotype to promote calcification [166].

Among the many biomolecules in EVs, ncRNAs are key regulators of their ability to mediate intercellular communication. MicroRNAs are regulatory molecules that inhibit protein expression, and they have been confirmed to be involved in the regulation of phenotypic transformation, senescence and calcification of VSMCs [27]. Several studies have shown that under pathological stimulation, different types of cell-derived EVs can regulate the phenotypic switch of VSMCs to promote VC by delivering miRNAs to recipient cells to upregulate the expression of Runx2 and activate various signaling pathways such as Wnt/β-catenin [167, 168]. And that transported miRNAs can further accelerate the development of VC through autophagy, oxidative stress, inflammation, immune response and other possible mechanisms [168]. A recent study has shown that functional molecule miR-23a-3p in EVs from atherosclerotic plaques can induce endothelial cell inflammation and propagate AS distantly through the circulation [169]. This study indicates that EVs are also regulated in the closed loop and affect neighboring cells, exacerbating disease progression. Similarly, there can be closed loop regulation between VMSCs through EVs.

In addition to the autocrine stimulation described above, EVs also play a key role in paracrine, such as the exchange of information between endothelial cells (ECs) and VSMCs [170–173]. And EVs mediate crosstalk between organs such as the bone-vessel axis [10] and so on. Furthermore, EVs derived from different cells also have potential improvement effects on cardiovascular diseases, such as progenitor cells(stem cells or mesenchymal cells)-EVs [174–176], BMSC-EVs [177], Cardiosphere-derived cells (CDCs)-EVs [178, 179] and so on. Therefore, we next focused on summarizing how EVs mediate communication between vascular wall cells (VSMCs-VSMCs, ECs-VSMCs, and Macrophages-VSMCs), bone-vascular, liver-vascular, and adipose-vascular under microenvironmental changes to regulate VC.

### Bone-vascular crosstalk

In recent years, studies have found that the skeleton is not only an "inert organ" that accepts nerve and humoral regulation but also an "endocrine organ" that participates in the regulation of the whole body. The bone acts on itself by autocrine and paracrine means, and also acts on extraskeletal organs by remote secretion via the circulatory system. Osteoporosis and vascular calcification are two pathological phenomena that seriously threaten the health of middle-aged and elderly people. Osteoporosis in the elderly is often accompanied by VC. With the aging of the body, the loss of calcium in bones increases, bone mass decreases, and osteoporosis gradually forms, while the calcium deposition in vascular wall tissues increases, resulting in increased rigidity and reduced compliance of blood vessels. This contradictory phenomenon is called the "calcification paradox" between bone and blood vessels [180]. Both VC and bone mineralization are actively regulated processes, and these two reviews discuss in detail the many similarities in pathophysiology and progression between osteoporosis and VC, and summarize clinical data supporting their interaction [95, 181]. However, whether osteoporosis drives VC or vice versa has not been established, and the specific mechanisms of their co-occurrence need to be further clarified. Previous studies have found that VC is accompanied by the expression of many bone metabolic proteins (osteopontin, adiponectin, bone morphogenetic protein 2[BMP2], osteoprotegerin, etc.) [18–21], suggesting the existence of a bone-vessel axis. It is believed that the bone-vessel axis may be regulated directly or indirectly by various hormones and physiological processes, but the specific mechanisms have not been fully elucidated.

#### Role of EVs in the regulation of the bone-vascular axis

With the development of aging and oxidative stress, the increase of miR-183 family (miR-96, miR-182, and miR-183–5 p) in bone matrix EVs (B-EVs) can inhibit the proliferation and osteogenic differentiation of Bone marrow mesenchymal stem cells (BMSCs) and induce stem cell aging [182–185]. In addition to the effects of B-EVs secretion on themselves, B-EVs also carry out long-distance cell-to-cell/organ-to-organ crosstalk. Surprisingly, a recent study by Wang et al. [10] suggests that EVs may help resolve this "calcification paradox". Osteoclastic bone resorption activity increases relative or absolute with bone aging and menopause, and B-EVs are found to be released from bone matrix into bone marrow during bone resorption and transported through blood circulation to vascular walls. It was found that EVs from aged bone matrix (AB-EVs) not only directly stimulated VSMC mineralization, but also found that the calcium content of AB-EVs was higher than that of YB-EVs(young bone-EVs), and increased serum calcium and inorganic phosphorus in both acute and chronic vascular calcification models, suggesting that AB-EVs can directly transport a large amount of calcium to circulation and increase phosphate in blood may be an important mechanism for AB-EVs to promote VC [10].

Another important mechanism is to reveal that AB-EVs mediates VC by transferring miR-483-5p and miR-2861, which are messengers of the "calcification paradox": miR-483-5p enrichment promotes adipogenesis (increased PPARγ expression) but not osteogenesis in BMSCs, and exacerbates calcification of VSMCs; it also stimulates RUNX2 expression and osteogenic transdifferentiation of VSMCs by transferring miR-2861 into the circulation and depositing it in blood vessels [10]. OC^YB^-CM (bone-resorption conditioned media from osteoclasts with young bone slices) and its EVs had no effect on VSMC calcification [10]. Intravenous or intramedullary injection of AB-EVs promoted bone fat imbalance and aggravated VC induced by vitamin D3 in young or old mice; it has also been found that the use of the bone resorption inhibitor alendronate (ALE) down-regulates the release of AB-EVs and reduces aging and ovariectomy-induced bone lipid imbalance and VC [10].

In a word, a new mechanism behind the "calcification paradox" related to age and menopause, namely the new mechanism of AB-EVs mediated "calcification paradox," provides a new idea for the prevention and treatment of osteoporosis and VC in the elderly, and further enriches the understanding of bone-vascular axis.

#### Kidney-gut-bone-vascular axis

In addition, intestinal microbiota plays a key regulator role in bone and cardiovascular homeostasis, and intestinal microbiota imbalance may induce osteoporosis and vascular calcification, which is envisioned as one of the pathogenesis involved in the bone-vascular axis. Chronic kidney disease is associated with a dysbacteriosis of the gut, which may contribute to bone and vascular disease in patients with CKD (the "kidney-gut-bone-vascular axis"), and potential mechanisms include: Decreased carbohydrate fermentation and increased protein fermentation result in gut-derived inflammation, deficiency of vit K and short-chain fatty acids (SCFAs) [186].

#### BMSCs-VSMCs crosstalk

Bone marrow mesenchymal stem cells (BMSCs) are pluripotent stem cells that can differentiate into a variety of cell types in vivo, such as: osteoblasts, chondroblasts, adipocytes, VSMCs, ECs and cardiomyocytes; and BMSCs and derived EVs have attracted great interest because they show great therapeutic potential as "miracle drugs." In previous studies, we have found that BMSC derived EVs may have the effect of relieving VC induced by high phosphorus (Pi), but the mechanism is not completely understood [187]. Guo et al. [187] found that BMSC-derived EVs attenuated VC induced by high Pi by changing the miRNA profile involving mTOR, MAPK and Wnt signaling pathways related to VC, suggesting that miRNAs may be key regulatory points for BMSC-EVs to inhibit VC. BMSC-EVs inhibit the osteogenic transdifferentiation and calcification of HA-VSMC by transferring miR-15a/15b/16 and inhibiting its common target gene nuclear factors of activated T cells 3 (NFATc3), thereby down-regulating the expression of osteocalcin (OCN) [188]. Liu et al. [189] proposed a novel mechanism that BMSC-EVs can also directly down-regulate NFAT5 by delivering miR-381-3p, improving apoptosis and VC in CKD-VC. Recent studies suggest that BMSC-EVs can regulate the NONHSAT 084969.2/NF-κB axis to inhibit Pi-induced VSMC transdifferentiation and calcification [190]. It also inhibits high phosphate-induced aortic calcification and improves renal function through the SIRT6-HMGB1 deacetylation pathway [191]. These studies suggest that BMSC-EVs may be a potential strategy in the treatment of CKD-VC.

Advanced glycation end products (AGEs) are proteins or lipids that become glycated after exposure to diabetes, which is the main cause of diabetic vascular complications, including diabetic vascular calcification [192]. Advanced Glycation End Product-Bovine Serum Albumin (AGEs-BSA) down-regulated and up-regulated the expression of miR-146a and thiotoxin-interacting protein(TXNIP) in VSMC, respectively, thereby increasing the production of ROS and promoting VSMC to differentiate into osteogenic phenotype [193]. However, miR-146a expression was increased in BMSC-EVs treated with AGEs-BSA, and EVs migrated into VSMC to inhibit AGEs-BSA-mediated VC by binding to its target, TXNIP [193]. Moreover, BMSC-EVs have an improved effect on cardiomyocyte function [177, 194], Intervertebral disc degeneration(IDD) and bone defect/damage [195].

In conclusion, it is further confirmed that EVs carrying multiple signaling molecules are important carriers for skeletal cells to regulate body functions, indicating the existence of bone-vascular axis regulation. The signaling pathway and gene expression of VSMCs are affected after uptake of EVs, which regulates calcification. And BMSCs-EVs showed great therapeutic prospects in VC.

### Liver-vascular crosstalk

The liver is the main organ of lipid metabolism and patients with metabolic-related fatty liver disease have varying degrees of abnormal lipid metabolism, leading to dyslipidemia, phenotypically elevated LDL cholesterol and triglycerides, which in turn accelerates the development of cardiovascular disease (CVD) [196]. Non-alcoholic fatty liver disease (NAFLD) is a disease prevalent in obesity and diabetes, caused by a combination of genetic and lifestyle factors, and shares common risk factors with CVD, including insulin resistance, central obesity, T2DM, dyslipidemia, and hypertension, among others. In recent years, the term "metabolic dysfunction associated fatty liver disease (MAFLD)" has been coined and is replacing the name "NAFLD" as it better reflects the underlying pathogenesis and cardiometabolic significance of NAFLD [197, 198].

In a large study of a young and middle-aged population, it was shown that AFLD and NAFLD are both metabolic liver diseases and that the early liver disease they cause, whether obese or non-obese, are strongly associated with and accelerate coronary artery calcification (CAC) [199]. The results of a recent large Korean cohort study showed a significant positive association between both NAFLD and MAFLD and the prevalence and incidence of CAC, with MAFLD being more strongly associated [200]. Several clinical studies have shown that NAFLD is strongly associated with CVD, and NAFLD patients are more likely to die from CVD than from liver-related disease [201–203]. NAFLD is not only limited to the progression of liver function deterioration but is becoming an independent risk factor for CVD [204].

In recent years, liver-derived sEVs have been found to play an important role in intercellular/interorgan communication [205]. The amount of hepatocyte-derived EVs release and the expression of miRNAs in their contents changed in different liver disease states [206]. For example, steatotic hepatocytes under NAFLD released more EVs, which mediates the development of inflammation, fibrosis and angiogenesis [207], and significantly alters the expression profile of miRNAs [208]. Steatotic hepatocyte-derived EVs promote endothelial cells inflammation and AS formation by delivering miRNA-1 to inhibits KLF4 and activates NF-κB, while anti-miR-1 attenuates this effect [208]. Furthermore, steatosis hepatocytes secreted sEVs abundant in novel-miR-7 in the circulation, which promotes hyperpermeability of coronary microvascular endothelial cells by directly regulating the lysosomal-associated membrane protein 1 (LAMP1)/Cathepsin B/NLRP3 inflammasome axis [209]. This may also be one of the mechanisms leading to the microvascular complications of NAFLD. Previously, the NLRP3 inflammasome has been shown to be involved in the regulation of VC [210–212].

These studies suggest that hepatocyte-derived EVs play an important role in the cross-talk between the liver and cardiovascular, providing new ideas about the mechanism of the link between NAFLD and CVD. It also suggests a possible link between hepatocyte-derived EVs and VC, and a liver-vascular axis may exist. However, basic studies of hepatocyte-derived EVs mediating cardiovascular calcification are scarce, and there is a need for more in-depth mechanistic exploration. Future studies devoted to EVs may not only contribute to the development of NAFLD but also largely reduce the occurrence of CVD and thus reduce the number of NAFLD patients who die from CVD.

### Adipose-vascular crosstalk

Adipose tissue (AT) is composed of a heterogeneous population of cells that regulate energy metabolism and immune responses. AT can divided into visceral adipose tissue and subcutaneous adipose tissue, among which abnormal deposits of visceral adipose tissue causing a higher incidence of cardiovascular disease [213]. The AT can act as an endocrine organ, secreting a variety of adipokines such as adiponectin, leptin, visfatin, omentin, etc. [214]. The AT-derived adipokines can mediate crosstalk between adipose tissue and cardiovascular via autocrine, paracrine and endocrine mechanisms [215]. For example, adiponectin inhibits beta-glycerophosphate (β-GP)-induced VC via the JAK2/STAT3 signaling pathway [216] and omentin-1 inhibits VC by activating AMPK and Akt signaling pathways [217]. In addition to the traditional soluble mediators described above, adipose tissue-derived EVs have also been shown to be an insoluble mediator that can regulate adjacent or distant target organs/cells.

Adipocyte derived EVs act as effective messengers between ADSCs and macrophages to maintain the metabolic and immune homeostasis of the body while driving a vicious cycle between M1 macrophages and hypertrophic adipocytes to cause immunometabolic imbalance [218]. Gan et al. [219] showed for the first time that miR-130b-3p was enriched in dysfunctional adipocyte-derived sEVs and inhibited multiple anti-apoptotic/cardioprotective molecules in cardiomyocytes, suggesting a novel mechanism for exacerbating MI/R injury in the diabetic heart. In addition, Human adipose tissue-derived mesenchymal stem cell-derived EVs (ASCs-EVs) could be taken up by endothelial cells, transfer miR-125a to endothelial cells and promoted angiogenesis by inhibiting DLL4 [220], and platelet-derived growth factor (PDGF) also can stimulate the secretion of ASCs-EVs and alter its protein composition to enhance the pro-angiogenic potential [221]. Perivascular adipose tissue (PVAT) is the adipose tissue that surrounds the aorta and releases various factors that regulate vascular function in a paracrine or autocrine manner [222]. PVAT causes proinflammation, the proliferation of VSMCs and vasoconstriction through the release of proinflammatory adipokines or cytokines that are associated with cardiovascular risk, can induce the development of AS, and are thought to be associated with VC [223]. Li et al. [224] found that PVAT-derived EVs and their abundantly loaded miR-221-3p could be taken up by neighboring VSMCs, leading to arterial remodeling and promoting the transformation of VSMCs from a contractile to a synthetic phenotype. Previously, miR-221 has been shown to be involved in the regulation of VC [225].

So far, the effect of adipose tissue-derived EVs on VC has not been fully explored, but the above studies show that AT-derived EVs may be involved in the regulation of VC as a new mediator, and we need to invest more research in the future.

### Crosstalk among vascular wall cells

#### VSMCs-VSMCs crosstalk

It is well known that VSMCs are the main cells in the tunica media of vascular wall and play a key role in the occurrence and development of VC [226]. It was found that under calcification-promoting conditions, VSMCs can be induced to produce calcification-promoting EVs, which act on adjacent normal VSMCs leading to a conversion to an osteogenic phenotype that promotes VC [17, 227], suggesting the existence of a closed-loop regulation.

##### Regulation of the release of EVs

Phingomyelin phosphodiesterase 3 (SMPD3, also known as Neutral sphingomyelinase 2,nSM2) has been shown to regulate the release of exosomes [228]. Increased SMPD3 expression under high extracellular calcium promotes the secretion of calcifying exosomes [17], and inhibition of SMPD3 expression by Barley-ß-glucans prevents VSMC calcification [229]. Lysosomal sphingolipids such as ceramide (CER) may promote mTOR activation on lysosomes, and lysosomal CER-mTOR signaling may prevent lysosomal fusion with MVBs within VSMCs and promote exosome secretion to induce arterial medial calcification (AMC) [230]. Increased CER production via the SMPD3 pathway may induce EVs biosynthesis to promote AMC [17]. And increased expression of the SMPD1 gene in the lysosomes of VSMCs [231]; deletion of the specific lysosomal ASAH1 gene leading to reduced TRPML1 channel-mediated Ca^2+^ release [232]; and absence of mucolipin-1 product of the Mcoln1 gene leading to abnormal localization of lysosomes [233], all of which can lead to reduced lysosomal-MVB interactions and increased MVBs fusion with the plasma membrane and EVs release, thereby promoting AMC. This may be a new molecular mechanism involved in the development of AMC. In a recent study, nano-sized hydroxyapatite (nHAp) was first demonstrated to promote the release of calcifying-exosomes through autophagy-lysosomal damage and accelerate VC [234]. Targeting this novel autophagy-lysosome-exosome pathway may help regulate the development of VC.

In addition, Kapustin et al. [235] found that circulating vitamin K–dependent coagulation proteins, PT in particular, a novel inhibitor of circulating vascular calcification and low circulating levels of PT in calcified patients, can bind to the surface of VSMC exosomes via PS and can also be loaded into exosomes by cellular internalization and recycling via the late endosome/multivesicular body (LE/MVB) compartment. The gradual loading of PT and PT activation products into exosomes inhibits procoagulant activity and nucleation site formation on the exosomes surface through interaction with Gla/PS, thus preventing exosome-mediated VC [235]. Thus, anticoagulation therapy with warfarin may enhance VC through impaired carboxylation of vitamin K–dependent coagulation factors delivered to VSMC-derived exosomes, which play a dual role in calcification and coagulation [235].

In this study, a novel mechanism for influencing intracellular calcium levels through calcium uptake by EVs as extracellular calcium was proposed: Extracellular Ca^2+^ entry into VSMCs is mediated by EVs through clathrin- and caveolin-mediated uptake, resulting in an increase in cytosolic Ca^2+^ [236]. Taken together, the above indicates that there are two mechanisms for calcium uptake through calcium channels: EVs-dependent and EVs-independent. Elevated cytosolic Ca^2+^ increases oxidative stress by promoting the expression of Nox5, and ROS can promote the release of EVs [236]. Contractile VSMCs are induced by PDGF and high Ca^2+^, and are dedifferentiated into synthetic VSMCs mediated by Nox5, which undergo a series of changes including apoptosis and osteogenic differentiation, secretion of more extracellular matrix (ECM) and EVs, all of which lead to increased calcification [236]. Studies have demonstrated for the first time that Nox5 is the molecular link between Ca^2+^, oxidative stress, and EVs release, and have confirmed Nox5 as a key regulator of VSMC phenotypic switching and calcification [236]. The risk of cardiovascular events is significantly increased in smokers, which is mainly caused by nicotine in cigarette components, which promotes VSMC calcification through α7 nAchR increasing intracellular Ca^2+^ and increasing Ca^2+^-dependent Nox5-induced ROS, thus increasing EVs release [164]. It was also found that the expression level of Nox5 in carotid artery of smokers was high and correlated with VC level [164]. In addition, pretreatment with vitamin K inhibited nicotine-mediated ROS production in VSMCs and decreased EVs release [164]. It was also found that the expression level of Nox5 in carotid artery of smokers was high and correlated with VC level. PCSK9 may contribute to the osteogenic phenotype of SMCs by mediating the release of more Ca^2+^-and ALP-rich EVs during VC associated with CKD [237]. High concentrations of Nε-Carboxymethyl-lysine (CML, a key active component of AGEs in serum) can significantly promote the release of SMCs-derived EVs and promote the recruitment of Sortilin to EVs, thereby exacerbating diabetic VC, while anti-sortilin treatment can significantly reduce VC caused by EVs [69]. Sortilin is a type I membrane glycoprotein encoded by SORT1 gene and belongs to vesicle sorting receptor. It participates in the loading and transportation of extracellular vesicles, which can transport the calcification-promoting protein TNAP into EVs [238], and can also promote the transportation of sortilin into EVs to form EVs with high mineralization capacity by forming homodimers containing intermolecular disulfide bonds [239], thus promoting VC. Sortilin plays an important role not only in the pathogenesis of cardiovascular diseases but also in metabolic diseases and cancers [240]. Furmanik et al. [241] demonstrated that endoplasmic reticulum (ER) stress mediated increased release of Grp 78-loaded EVs to promote VSMC calcification, but not through apoptosis; Warfarin can induce endoplasmic reticulum stress through PERK-ATF4 pathway, increase the release of EVs, and promote calcification [241].

##### Regulation of bioactive molecules in EVs

In addition, EVs can also transfer functional bioactive molecules (such as miRNA, proteins, etc.) to regulate the development of VC. Regulation of VC by changing protein content in EVs, For example, the GFOGER peptide (a specific six-amino-acid repeat in COL1 sequence) reduces VC by modulating the content of proteins associated with the osteogenic phenotype in VSMC-derived EVs (e.g. ANK-binding kinase 1 and casein kinase II) and inhibiting osteogenic transdifferentiation of VSMCs [242]. Previous studies have shown that GFOGER peptide partially inhibits VC by preventing EVs-COL1 interaction [243].

Moreover, more attention has been paid to miRNAs, which can be regulated by changing the amount of miRNAs in EVs. For example, the miRNA profile in EVs derived from calcified VSMCs was markedly altered, with 987 and 92 miRNAs significantly upregulated and downregulated [244]. It has been reported that Curcumin (CUR, a natural polyphenol compound with lipid-lowering, anti-inflammatory and antioxidant effects on cardiovascular system) may inhibit VC by increasing the content of miR-92b-3p loaded in VSMC-derived EVs and reducing the expression of its target KLF4, thus affecting the expression of RUNX2 [245]. In a recent study, Melatonin attenuated osteogenic differentiation and senescence of VSMC or calcified vascular smooth muscle (CVSMCs) by upregulating miR-204/miR-211 in EVs secreted by VSMCs or CVSMCs [71].

**Fig. 3:**
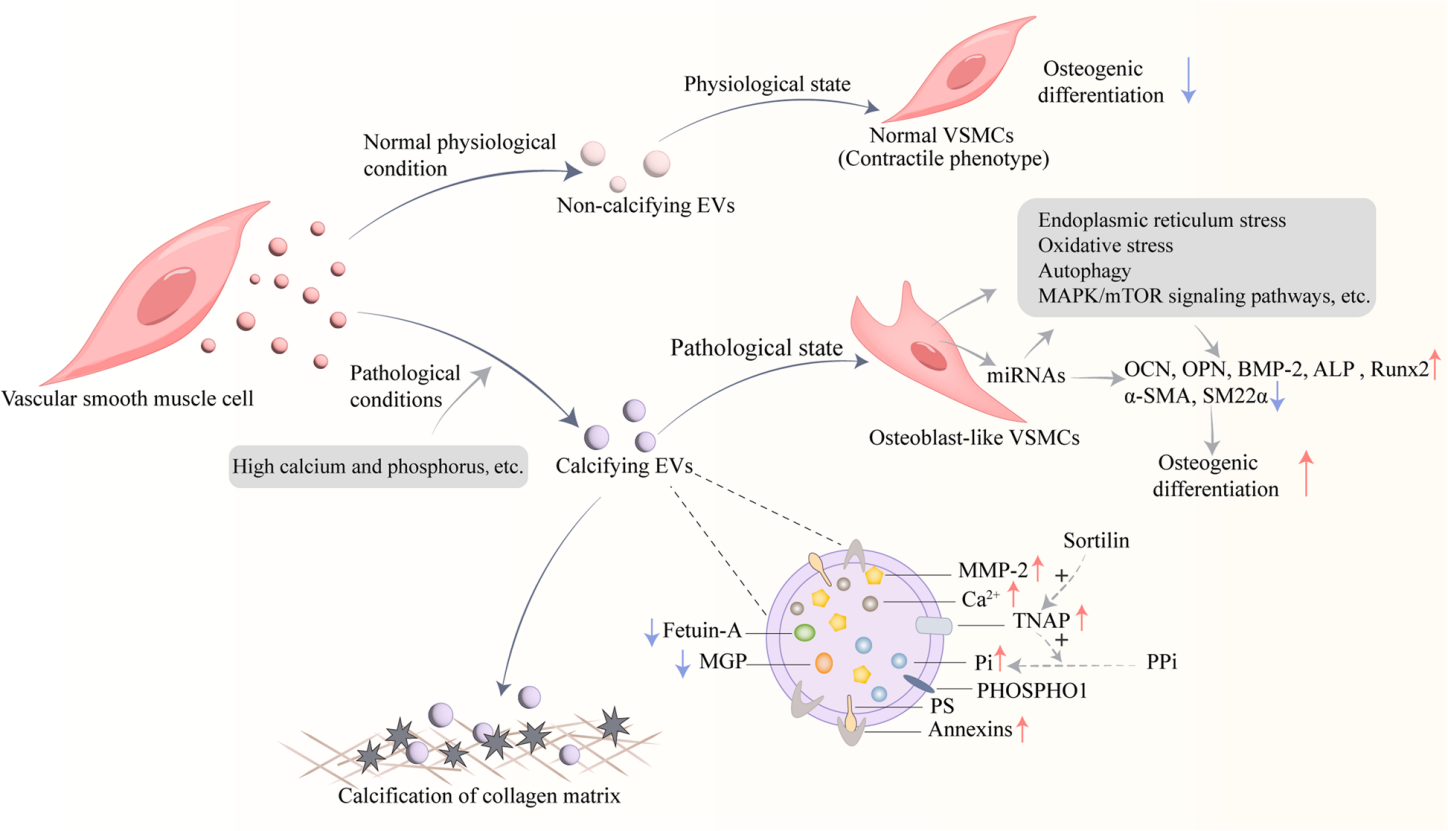
Potential mechanism of VSMCs-derived EVs regulating osteogenic differentiation and calcification of VSMCs. VSMCs spontaneously releases EVs under physiological conditions, which are loaded with the calcification inhibitors MGP and Fetuin-A. Pathological stimuli can alter the biological characteristics of EVs, promoting the formation and release of EVs with calcification potential. Calcified EVs contain less MGP and Fetuin-A and more MMP-2, annexins, TNAP, Ca^2+^ and Pi. When calcified EVs are released into the extracellular matrix (ECM) and interact with it to promote the release and aggregation of hydroxyapatite crystals and form microcalcifications. TNAP may shift the Pi/PPi balance toward calcification by hydrolyzing the calcification inhibitor inorganic pyrophosphate (PPi) and generating inorganic phosphate (Pi) required for mineralization, which is critical for EVs mediated hydroxyapatite formation. Sortlin regulates TNAP entry into EVs, increasing the calcification potential of EVs. EVs contain another phosphatase, PHOSPHO1, which acts as an alternative supplier of Pi, and plays a role in the initiation of matrix mineralization. Calcified EVs formed under pathological stimuli regulate VC through the following mechanisms:(1) promoting extracellular mineral deposition;(2) promoting osteogenic phenotype transformation of VSMC;(3) transferring microRNAs between cells; and (4) regulating signaling pathways

In conclusion, microenvironment changes (high calcium, ROS, endoplasmic reticulum stress, lysosome activity and autophagy-lysosomal damage, etc.) can promote VC by increasing the release of VSMC-EVs, and carry more abundant pro-calcification substances. They can mediate mineral deposition to initiate calcification, as well as propagate calcification through the transfer of bioactive molecules (such as miRNAs and proteins) or regulation of signal transduction (Fig. 3). Altering the nature and biogenesis of VSMC-derived EVs may be an effective strategy to limit vascular calcification. These studies provide strong evidence that EVs regulate VC through information exchange between VSMCs-VSMCs. And in the future treatment and prevention of VC has a vital role.

#### ECs-VSMCs crosstalk

Vascular endothelial cells (ECs) are also the main cells involved in vascular system diseases [246] and are located in the intima, which means that they act as "frontrunners" that can be directly stimulated by pathological triggers in the circulating blood (such as hyperglycemia, hyperphosphatemia, uremic toxins) [247–249]. It has been proved that VC often occurs in the tunica media in patients with diabetic mellitus or CKD [250]. However, how does high glucose and high phosphorus in circulation transfer from the intima to the media and thus affect calcification/aging of VSMC that are not contact with blood directly? Therefore, most of the studies on arterial media calcification focus on ECs in recent years. ECs can synthesize and secrete several Gas signals (e.g., H_2_S, NO, and CO) and small molecule peptides (e.g., CNP, ADM, PTH, and ET), and so on, which directly affect VSMCs to promote VC formation and development [251]. Increasing evidence suggests that ECs are involved in VC through the following mechanisms: endothelial dysfunction [252, 253], endothelial-mesenchymal transition (EndMT) [23], autocrine/paracrine pathways [254], angiogenesis [255], and mechanotransduction [256]. Importantly, a series of studies have proved that ECs can also communicate with VSMCs through EVs. For example, CXCR6 promote calcification by downregulating miR-29b in aortic endothelial cell (AEC)-derived exosomes [257]. ECs-derived EVs also can regulate signal transduction pathways, such as triggering a pro-inflammatory, hypertrophic and senescent phenotypes in VSMCs through a mechanism involving high-mobility group box proteins (HMGB) [170].

Inflammatory stimuli and CKD and/or senescence-induced endothelial damage lead to increased BMP2 expression in HUVEC and increase the release of their microvesicles loaded with abundant Ca^2+^ and BMP2 to promote osteogenic transformation and calcification of VSMCs [258]. The study further suggests that the number of circulating microvesicles in plasma increases with age and that these microvesicles may be produced by aging ECs to induce VC [162]. Several studies have demonstrated that the pro-calcific potential of aged HUVEC-derived microvesicles is related to the substances carried: carries more calcium; can carry extra calcium-binding proteins (such as annexin A2 and A6); more bone related protein (BMP2) [18, 109, 162]. These studies demonstrate that aging is inextricably linked to ECs-EVs. Next, we have summarized how ECs, when stimulated by high glucose and high phosphorus in circulation, regulate VSMCs through EVs.

##### Crosstalk in CKD-related VC

Some studies have shown that ECs-EVs are closely related to CKD-VC. Guava FACS analysis of EV from dialysis patients at T0 showed that only endothelium-derived EVs were up-regulated among most circulating EVs derived from monocytes/macrophages, platelets and endothelial cells in CKD patients compared with healthy recipients, indicating that endothelial cells are the main participants in circulating EVs in CKD patients [259]. A recent study suggests that by transcriptomic analysis of circulating sEVs from CKD mice models as well as from CKD patients, possibly from endothelial cells, the calcification-protecting miRNAs that target VEGFA signaling in CKD-driven vascular calcification are lacking: miR-16-5p, miR-17-5p, miR-20a-5p, and miR-106b-5p [67]. And the sEVs biogenesis system inhibitor GW4869 [63–65], was used to ameliorate aortic VC in CKD model mice [67]. The areas under the ROC curves for miR-16-5p, miR-17-5p, miR-20a-5p and miR-106b-5p, which further predicted aortic vascular calcification in CKD patients, were 0.7630, 0.7704, 0.7407 and 0.7704, respectively, suggesting that the lower expression levels of these four miRNAs could predict abdominal aortic calcification [67]. Alique et al. [260] found in vitro that uremic toxin such as indoxyl sulfate (IS) induces stress, premature senescence in endothelial cells and increases the release of microvesicles to cause osteogenic transformation of VSMCs and an inflammatory response that promotes the development of VC. In addition, uremic toxins-treated senescent endothelial cell-derived EVs, and uremic rat plasma-derived EVs (hereinafter referred to as uraemic EV, EV^UR^) promote calcium-and phosphorus-induced osteogenic differentiation and calcification of VSMC [172]. The mechanism has been demonstrated that EV^UR^ accelerates and increases pro-calcifying-mediated (CM) expression of runx2, osterix, and osteocalcin, and decreases gene expression of sm22α in VC; also increased protein expression of the phosphate transporter PIT-1 in VSMCs and induced phosphorylation of AKT and ERK [172]. Reducing the expression of PiT-1 and inhibiting AKT and ERK signaling pathways alone can block the calcification-promoting effect of EV^UR^ [172].

In addition, EV^UR^ significantly down-regulated miR-143 and miR-145, whereas transfection of the mimetics of miR-143/145 alone into EV^UR^ blocked the pro-calcific effects of EV^UR^ [172]. And the combined use of miR-143 and miR-145 mimics was similar to the combined use of miR-221 and miR-222 inhibitors, which could more significantly block the aggravating effects of EV^UR^ on CM-induced calcification [172]. For Hp-EC-EVs (hyperphosphatemia-induced-endothelial extracellular vesicles) miRNA-seq analysis showed that miRNAs expression were changed: 12 down-regulated miRs (has-miR193b-5p, hsa-miR-941, hsa-miR-99b-5p, hsa-miR-365a-5p, hsa-miR-30c-2-3p, hsa-miR-30a-3p, hsa-miR-30a-5p, hsa-miR-486-5p, hsa-miR-7706, hsa-miR-10a-5p, hsa-miR-10b-5p and including hsa-miR-143-3p as confirmed above) and an up-regulated hsa-miR-3182, suggesting that these miRs may be potential markers for CDK-VC; KEGG analysis of Top-20 signaling pathways showed that calcium signaling pathway, cAMP signaling pathway, and ABC transporters may be closely related to VC [261]. A recent study also shown that miR-670-3p released from ECs-EVs plays an important role in regulating VC and may be a potential target for arterial calcification in patients with CKD [68]. These studies show that ECs and their derived EVs play a key role in CKD and aging, and help to elucidate the mechanism of CKD-VC from the perspective of ECs-EVs.

##### Crosstalk in diabetes-related VC

AGEs activate ECs by binding to its receptor RAGE, and then induce the production of reactive oxygen species (ROS), which can damage ECs and ultimately lead to apoptosis [262, 263]. The interaction between ECs and VSMCs mediated by EVs have also received considerable attention in the HG environment. One study showed that EVs derived from high glucose-induced HUVEC(HG-HUVEC-EVs) delivered Notch3(a key regulator of VSMC proliferation and phenotypic transformation) to VSMC through mTOR signaling pathway, which promoted VSMC calcification/aging [173]. In another study, HG-HUVEC-EVs contain functional VCAN, which may promote VSMC calcification/senescence by regulating mitochondrial function [264]. In addition, Li et al. [264] revealed that HG-HUVEC-EVs also increased the levels of MDA(which is produced by lipid peroxidation of polyunsaturated fatty acids and MDA levels increases under oxidative stress) and LDH(intracellular enzyme existing in cytoplasm, the leakage of which from cells is considered to be an important indicator of cell damage) and significantly reduced the activity of SOD (an important antioxidant enzyme involved in antioxidant defense) in VSMCs. Surprisingly, unlike previous studies showing that AGEs are harmful to vascular cells, the latest study by Guo et al. [70] shows that AGEs can inhibit diabetic media calcification by stimulating HUVEC to secrete miR-126-5p-rich sEVs, targeting BMPR1 and thus blocking smad1/5/9 signaling pathway. Although EVs and their cargo may be potential therapeutic targets for diabetic calcification, this contradiction means that further in vivo studies and clinical applications are needed to assess the therapeutic value of EVs in patients with VC.

##### ECs-other cells crosstalk

Some studies have shown that the EVs-mediated crosstalk between other cells and ECs/other cells also mediates cardiovascular calcification. For example, valvular interstitial cells (VICs) are the most abundant cells in the aortic valve and have the ability to convert to an osteogenic phenotype and calcify [265, 266]. Although the mechanism of this process has not been fully elucidated, VICs can inhibit VEC (valvular endothelial cell) EndMT and osteogenesis, suggesting that valve homeostasis is maintained by appropriate VIC-VEC interactions [267]. In calcific aortic disease, VICs secrete pro-calcific EVs to interact with ECs and thereby remodel the ECM [268]. Annexins II, V, and VI are up-regulated in pro-calcific VICs-derived EVs, and these annexins mediate calcium inward flow into the EVs [109]. Elevated circulating calcium and phosphorus levels in ESRD patients may increase the levels of annexin VI and calcium loaded in VICs-EVs to promote aortic valve calcification [269]. Telocytes (TCs) are novel mesenchymal cells that are widely distributed in the extracellular matrix of any tissue and can participate in cellular communication through direct homo- and heterocellular junctions or release EVs [270, 271]. Interestingly, Yang et al. [272] showed for the first time that TCs-derived EVs co-cultured with calcified VICs could improve aortic valve calcification through the miR-30b/Runx2/Wnt/β-catenin axis. These studies suggest that calcified EVs secreted by VICs may be mediators of heart valve calcification, and may also be mediators of VICs-VECs interaction mediating valve calcification. Aortic valve calcification remains an unresolved clinical problem and requires more attention to the mechanistic role of VICs-EVs in the development of aortic valve calcification.

In conclusion, EVs perform critical functions in intercellular communication by transferring their bioactive substances to receptor cells or activating signaling pathways in target cells. New perspective of ECs-derived EVs reveals the relationship between endothelial cells and vascular calcification, and can better understand the complex mechanism of how some substances induce VC development through ECs-VSMCs crosstalk without direct contact with VSMCs.

#### Macrophages (Immune Cells)-VSMCs crosstalk

Macrophages are a type of innate immune cell that reside in the intima and adventitia of blood vessels [273]. Their primary function is to clear cellular debris, lipids, and other foreign substances from within the blood vessels, thereby maintaining vascular health [274]. And remarkable plasticity and functional heterogeneity are important features of macrophages [275]. Macrophages are involved in almost all the stages of vascular calcification, and different subtypes of macrophages play different roles. M1 macrophages (pro-inflammatory) produce pro-inflammatory factors such as IL-1β, IL-6 and TNF-α to promote osteogenic transdifferentiation of VSMCs [276–278]. Similarly, products of oxidative stress such as ROS can induce a switch in VSMC phenotype toward pro-calcification [279]. M1 macrophage can directly release oncostatin M (OSM) to promote the differentiation of VSMCs into osteoblastic phenotypes through the JAK3-STAT3 pathway [280]. In contrast to M1 macrophages, M2 macrophages are anti-inflammatory and are thought to be protective against VC. M2 macrophages express low or no pro-inflammatory mediators (e.g., TNFα) and highly express anti-inflammatory mediators (e.g., IL-10 and TGF-β) [281]. In addition, M2 macrophages can increase the concentration of the calcification inhibitor pyrophosphate/ATP to inhibit hydroxyapatite crystal nucleation [282]. In plaque progression, M1 macrophages may promote microcalcification formation by consistently expressing pro-inflammatory cytokines such as TNF-α, IL-6 and MMPs, whereas these macrophages transform into M2 macrophages in plaque regression and secrete of anti-inflammatory cytokines such as IL-10 and TGF-β may promote macrocalcification [283, 284]. Oncostatin M and statins may facilitate plaque regression by enhancing M2 polarization of macrophages, leading to plaque macrocalcification and stabilization [283]. Collectively, M1 and M2 have nearly opposite effects in regulating the development of VC, suggesting that using the plasticity of macrophages to impel a polar shift in different subsets could offer a new breakthrough for calcification regression. Macrophages can promote VC through various mechanisms such as release of reactive oxygen species, pro-inflammatory cytokines, polarization drift of macrophages, phenotypic transformation of macrophages and matrix vesicles. The potential mechanisms of macrophage involvement in calcification are evaluated in these two reviews [8, 285].

Molecular imaging shows coexistence of macrophages with microcalcifications [286]. In inflammation-mediated calcification, inflammation precedes microcalcification and is mediated by the release of EVs from macrophages, and macrophage-derived EVs may be a bridge to mineral deposition in these tissues. Furthermore, it was found that macrophage-derived EVs also play a role in crosstalk. Therefore, we next summarize the role of macrophage-derived EVs in mediating mineral deposition and acting as mediators of communication.

##### Macrophage-derived EVs mediate mineral deposition to form microcalcifications

S100A9-positive macrophages were found in early atherosclerotic plaques and it was confirmed that S100A9 is associated with vascular inflammation and calcified EVs [287]. To further characterize macrophage-derived EVs as having a potent role in calcification, it is proposed that pro-inflammatory S100A9, a calcium-binding protein identified as a biomarker of acute cardiovascular events [288], has a key role in macrophage EVs-mediated microcalcification. S100A9 was found to interact with annexin V to form the PS-Annexin V-S100A9 membrane complex as a nucleation center for hydroxyapatite [288]. Calcium/Phosphorus stimulation promoted macrophages to release MVs enriched in calcium, ALP, S100A9 and annexin V, which directly promoted the formation of microcalcifications [289].

Macrophage-derived EVs-mediated microcalcification has been shown to be associated with the development of diabetic vascular calcification [290]. Macrophage S100A9 also plays a key regulatory role in diabetic vascular calcification. Increased secretion of S100A9 and increased expression of receptor for advanced glycosylation end-products (RAGE) protein in human primary macrophages under high glucose (25 mmol/L) conditions; the S100A9-RAGE signaling pathway promotes macrophage expression of proinflammatory and osteogenic factors and induces the release of calcified EVs from proinflammatory macrophages; and revealed a novel mechanism by which the S100A9-RAGE axis may regulate proinflammatory and pro-osteogenic macrophage activation through crosstalk between NRF-2 (nuclear factor 2 erythroid related factor 2) and NF-κB (nuclear factor-κB) pathways [290]. These findings suggest that macrophage S100A9 is a key regulator of diabetic-mediated vascular calcification. Modulation of S100A9 and Annexin V may inhibit the formation of hydroxyapatite nucleation and unstable microcalcifications in macrophage-derived MVs [289].

##### EVs transfer bioactive molecules or trigger signaling pathways

Pro-inflammatory macrophage-derived EVs deliver miR-241, which is associated with inflammatory response, to VIC and downregulate TWIST1 expression to promote aortic valve calcification (AVC) [291]. Recent studies have confirmed that miR-32 is an important molecule for macrophage-derived EVs to inhibit VSMC autophagy to promote T2D VC [292]. In addition, upregulation of galectin-3 (a marker of cardiovascular disease) in macrophages promotes migration of VSMC-derived EVs to the intima and induces the formation of diabetic vascular intimal calcification [293]. Yaker et al. [294] recently showed that lipopolysaccharide-treated macrophage-derived EVs induced inflammation and oxidative stress in VSMCs, thereby promoting the development of VC.

To date, studies of immune cell-derived EVs in inflammation-mediated cardiovascular calcification have focused on macrophages in the innate immune response. However, other innate immune cells (such as Dendritic cells, Mast cells, Neutrophils and Natural Killer cells) and adaptive immune-mediated inflammation related vascular calcification studies will also be an exciting area for future and provide a deeper understanding of inflammation-mediated calcification. Although their role in cardiovascular calcification remains poorly understood, and crosstalk between macrophages and other immune cells has rarely been described [295]. Therefore, an in-depth study of the molecular mechanisms of immune cell-derived EVs in response to VC is warranted.

**Fig. 4:**
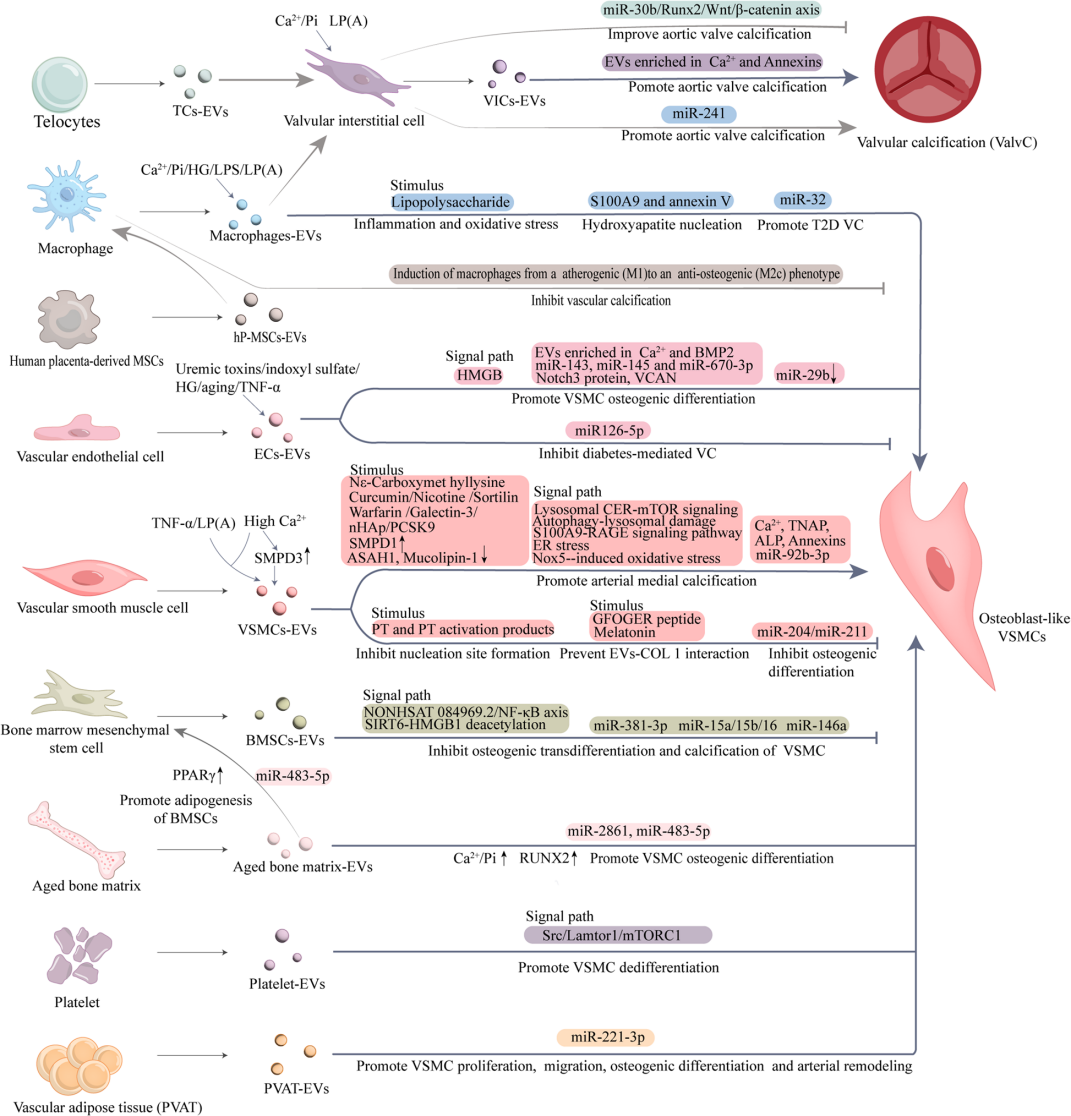
Extracellular vesicle-mediated intercellular crosstalk in vascular calcification. Different cell-derived EVs mediate communication between cells, such as by delivering miRNAs and osteogenic-associated proteins, or by regulating signaling pathways and so on, thereby regulating VC

In summary, the relationship between EVs-mediated intercellular communication was briefly summarized in Fig. 4. And the summary of bioactive cargo in EVs derived from different cells and their regulatory pathway in Table 1.

**Table 1:** Extracellular vesicle-mediated intercellular crosstalk regulates the occurrence and development of vascular calcification

Extracellular vesicle orgin	Donor cell-Recipient cell	Mechanisms	Effects on vascular calcification	Refs
Aged bone matrix (AB-EVs)	Bone cell-VSMCs	•Increased serum calcium and inorganic phosphorus •Transfer miR-2861	•Stimulated Runx2 expression and osteogenic transdifferentiation of VSMCs •Aggravated VC induced by vitamin D3 (VD3)	[10]
BMSCs-EVs	BMSCs-VSMCs	Altered miRNA profiles of mTOR, MAPK and Wnt signaling pathways associated with VC	Alleviated high phosphorus (Pi)-induced VC	[187]
BMSCs-EVs	BMSCs-VSMCs	Regulated of the NONHSAT 084969.2/NF-κB axis	Inhibited pi-induced VSMC transdifferentiation and calcification	[190]
BMSCs-EVs	BMSCs-VSMCs	By SIRT6-HMGB1 deacetylation pathway	Inhibited high Pi-induced aortic calcification	[191]
BMSCs-EVs	BMSCs-VSMCs	Transfer of miR-381-3p downregulates NFAT5	Improved apoptosis and VC in CKD	[189]
BMSCs-EVs	BMSCs-VSMCs	Transfer of miR-15a/15b/16 inhibit target NFATc3 and further down-regulates OCN expression	Inhibited osteogenic transdifferentiation and calcification of VSMC	[188]
BMSCs-EVs	BMSCs-VSMCs	Transfer of miR-146a and binding to its target TXNIP	Inhibited AGEs-BSA-mediated VC	[193]
VSMC-exosomes	VSMCs-VSMCs	•Increased SMPD3 expression under high extracellular calcium promotes the secretion of calcifying exosomes •Increased CER production via the SMPD3 pathway may induce EVs biosynthesis	Promoted arterial medial calcification (AMC)	[17]
VSMC-exosomes	VSMCs-VSMCs	Lysosomal CER-mTOR signaling	Promoted arterial medial calcification	[230]
VSMC-exosomes	VSMCs-VSMCs	Increased expression of the SMPD1 gene in the lysosomes of VSMCs	Promoted arterial medial calcification	[231]
VSMC-exosomes	VSMCs-VSMCs	Deletion of the specific lysosomal ASAH1 gene leading to reduced TRPML1 channel-mediated Ca^2+^ release	Promoted arterial medial calcification	[232]
VSMC-exosomes	VSMCs-VSMCs	Absence of mucolipin-1 product of the Mcoln1 gene leading to abnormal localization of lysosomes	Promoted arterial medial calcification	[233]
VSMC-exosomes	VSMCs-VSMCs	Nano-sized hydroxyapatite (nHAp) promoted the release of calcifying-exosomes through autophagy-lysosomal damage	Accelerated VC	[234]
VSMC-exosomes	VSMCs-VSMCs	PT and PT activation products inhibit nucleation site formation	Prevented exosome-mediated VC	[235]
VSMC-EVs	VSMCs-VSMCs	Promoted the release of calcified EVs rich in Annexins under calcified medium containing pro-inflammatory Lp(A)	Aggregated in the ECM to mineralize to form microcalcifications and combine to form macrocalcifications	[165]
VSMC-EVs	VSMCs-VSMCs	Elevated cytosolic Ca^2+^ can promote the release of EVs by increasing the expression of Nox5 and increasing oxidative stress	Promoted osteogenic differentiation, apoptosis and calcification of VSMC	[236]
VSMC-EVs	VSMCs-VSMCs	Nicotine promoted EVs by increasing intracellular ca^2+^ and ca^2+^-dependent Nox5-induced ROS via α7 nAchR	Promoted VSMC calcification	[164]
VSMC-EVs	VSMCs-VSMCs	PCSK9 induced the release of Ca^2+^-and ALP-rich EVs	Promoted osteogenic phenotype of VSMCs in CKD-VC	[237]
VSMC-EVs	VSMCs-VSMCs	•High concentrations of Nε-Carboxymet hyl-lysine promoted the release of EVs •Promoted the recruitment of Sortilin to EVs	Exacerbated diabetes-related VC	[69]
VSMC-EVs	VSMCs-VSMCs	Sortilin transport TNAP into EVs	Promoted vascular calcification	[238]
VSMC-EVs	VSMCs-VSMCs	Promoted the transportation of sortilin into EVs to form EVs with high mineralization capacity by forming homodime rs containing intermolecular disulfide bonds	Promoted vascular calcification	[239]
VSMC-EVs	VSMCs-VSMCs	•Endoplasmic reticulum (ER) stress promoted the release of Grp 78-loaded EVs •Warfarin increased EVs release by inducing ER stress through PERK-A TF 4 pathway	Promoted VSMC calcification	[241]
VSMC-EVs	VSMCs-VSMCs	GFOGER peptide regulated protein content associated with osteogenic phenotype in EVs	Inhibited osteogenic transdifferentiation of VSMCs	[242]
VSMC-EVs	VSMCs-VSMCs	GFOGER peptide prevent EVs-COL 1 interaction	Inhibited VC	[243]
VSMC-EVs	VSMCs-VSMCs	Curcumin increased the content of miR-92b-3p loaded in EVs and reduced the expression of its target KLF4	Decreased the expression of Runx2 to inhibit calcification	[245]
VSMC-EVs	-	Upregulation of galectin-3 in macrophages promotes migration of VSMC-EVs to the intima	Induced the formation of diabetic vascular intimal calcification	[293]
VSMCs/calcified vascular smooth muscle cells (CVSMCs)-EVs	VSMCs-VSMCs	Melatonin up-regulated miR-204/miR-211 in EVs	Decreased osteogenic differentiation and senescence of VSMCs/CVSMCs	[71]
CVSMCs-EVs	CVSMCS-VSMCs	Induced cell signaling changes and phenotypic alteration of recipient VSMC	Accelerated vascular calcification	[227]
Endothelial cells-EVs	ECs-VSMCs	•High glucose-induced EVs could increase the levels of MDA and LDH and decrease the activity of SOD in VSMCs •High glucose-induced EVs contain functional VCAN	Mitochondrial dysfunction and osteogenic phenotypic transformation of VSMC	[264]
Endothelial cells-EVs	ECs-VSMCs	EVs through mechanisms involving high-mobility group box proteins (HMGB)	Induced a pro-inflammatory, hypertrophic and senescent VSMC phenotype	[170]
Endothelial cells-EVs	ECs-VSMCs	Increased the release of microvesicles loaded with abundant Ca^2+^ and BMP2	Promoted osteogenic transformation and calcification of VSMCs	[258]
Endothelial cells-EVs	ECs-VSMCs	EVs lack miR-16-5p, miR-17-5p, miR-20a-5p and miR-106b-5p that synergistically target VEGFA	Promoted CKD-induced VC	[67]
Endothelial cells-microvesicles	ECs-VSMCs	Uremic toxin such as indoxyl sulfate (IS) increased the release of microvesicles	Promoted osteogenic transformation of VSMCs and inflammatory response	[260]
Endothelial cells-EVs	ECs-VSMCs	•Uremic toxins-treated EVs (EV^UR^) increased expression of runx2, osterix, and osteocalcin •EV^UR^ increased protein expression of the phosphate transporter PIT-1 in VSMCs and induced phosphorylation of AKT and ERK •EV^UR^ significantly down-regulated miR-143 and miR-145	Promoted calcium-and phosphorus-induced osteogenic differentiation and calcification of VSMC	[172]
Endothelial cells-EVs	ECs-VSMCs	miR-670-3p	Inducted CKD-mediated calcification	[68]
Endothelial cells-EVs	ECs-VSMCs	High glucose induced EVs to transfer Notch3 protein to VSMC via mTOR signaling pathway	Promoted VSMC calcification/aging	[173]
Endothelial cells-EVs	ECs-VSMCs	AGEs stimulate the secretion of miR126-5p-rich sEVs, targeting BMPR1 to block the smad1/5/9 signaling pathway	Inhibited diabetic media calcification	[70]
Aortic endothelial cell (AEC)- exosomes	-	Downregulated of miR-29b in exosomes by CXCR6 leads to dysregulation of MMPs and collagens	Promoted vascular calcification and remodeling to reduce aortic compliance	[257]
VICs	-	Promoted the release of calcified EVs rich in Annexin proteinin under calcified medium containing pro-inflammatory Lp(A)	Promoted valve calcification	[165]
VICs	-	Hypercalcemia may increase the levels of annexin and calcium loaded in EVs	Pomoted aortic valve calcification	[269]
Telocytes (TCs)-EVs	TCs-VICs	By miR-30b/Runx2/Wnt/β-catenin axis	Improved aortic valve calcification	[272]
Macrophage-matrix vesicles (MVs)	-	•Ca/Pi-stimulation promoted the release of mineralizing MVs from macrophages with increased calcium loading of MVs and increased alkaline phosphatase activity •MVs enriched in S100A9 and annexin V promote hydroxyapatite nucleation	Promoted the formation of microcalcification	[289]
Macrophage-EVs	-	High glucose induced the release of calcified EVs from proinflammatory macrophages via S100A9-RAGE signaling pathway	Promoted diabetic vascular calcification	[290]
Macrophage-EVs	Macrophages-VICs	Deliver miR-241 and downregulate TWIST1 expression	Promoted aortic valve calcification (AVC)	[291]
Macrophage-EVs	Macrophages-VSMCs	Delivery of miR-32 to inhibit VSMC autophagy	Promoted T2D VC	[292]
Macrophage-EVs	Macrophages-VSMCs	EVs from lipopolysaccharide-treated induce inflammation and oxidative stress in VSMCs	Accelerated VC process	[294]
Platelet-EVs	Platelet-VSMCs	EVs may via Src/Lamtor1/mTORC1 signaling pathway	Promoted VSMC dedifferentiation	[296]
Perivascular adipose tissue (PVAT)-EVs	PVAT-VSMCs	Transfer of miR-221-3p	Promoted VSMCs proliferation and migration, to the synthetic phenotype of transdifferentiation and lead to arterial remodeling	[224]

## Circulating EVs as potential predictors in cardiovascular events

Circulating EVs include EVs from platelets, endothelial cells, neutrophils, monocytes, lymphocytes, erythrocytes, and their precursor cells. With the occurrence of CVD, circulating EVs of platelets and endothelial cells, among other sources, are significantly higher than in normal persons [297, 298]. Oggero et al. [299] during 3.5 years of follow-up (median) showed that in a population at risk for major adverse cardiovascular event or death (MACE) and receiving atorvastatin therapy (compared with controls and placebo, respectively), the biology of plasma-derived EVs was significantly different in size, level, and surface characteristics. MACE patients had preceding higher levels of CD14^+^ and CD14^+^/CD41^+^EVs, and higher CD14^+^ extracellular vesicles were associated with a 3.7-fold increased risk of MACE on matched analysis [299]. Patients treated with atorvastatin had both reduced size of extracellular vesicles and the proportion of CD146^+^ extracellular vesicles [299]. Kanhai et al. [300] conducted a large prospective cohort study including 1060 patients with CVD and found that elevated levels of Cystatin C, Serpin F2 and CD14 in plasma-derived EVs were associated with an elevated risk of future vascular events and death in patients with clinically manifest vascular disease. And CD144^+^ in endothelial cells-derived EVs as an independent predictor of cardiovascular event occurrence may contribute to risk stratification of coronary heart disease in populations with coronary risk factors [301]. In addition, several studies have found that circulating miRNAs can also predict the risk of cardiovascular events. Patients with low expression of miR-126 and miR-199 have a high incidence of cardiovascular events, and ECs-derived EVs are the major source of circulating miR-126 and miR-199, while platelet-derived EVs are another pathway of miR-126 origin [302, 303]. Platelet-derived EVs are potent regulators of immune cells and have shown strong immunomodulatory capacity on VSMCs, leading to phenotypic changes, migration and proliferation, which may accelerate the progression of vascular diseases [304, 305]. It has been shown that platelet-derived EVs promote VSMC dedifferentiation may via Src/Lamtor1/mTORC1 signaling pathway [296]. A recent study, Koide et al. [67] found that low expression levels of miR-16-5p, miR-17-5p, miR-20a-5p, and miR-106b-5p in circulating sEVs, possibly derived from endothelial cells, in patients with CKD predicted calcification of abdominal aorta. Compared with histopathology, blood and body fluid-based EVs tests are highly acceptable to patients and facilitate monitoring and reflecting the overall condition of the disease. Compared with traditional markers, EVs have the advantages of targeting, packaging greater information and easy storage, etc. Therefore, EVs-related biomolecules are circulating markers with considerable clinical diagnostic value.

## Treatment strategy of vascular calcification based on "crosstalk"

EVs also have a "disease-curing" effect. EVs can improve VC by mechanisms such as acting as direct targets or transferring bioactive substances between cells or modulating signaling pathways.

Chen et al. [306] showed that the calcium channel blocker verapamil inhibited the production and activity of calcifying EVs and also reduced the calcifying capacity of COL 1 to prevent ECM mineralization. BGP-15 is a new anti-diabetic drug candidate that increased MGP content and decreased Annexin A2 content in EVs and prevented calcium deposition in the ECM [307]. Alendronate (ALE), a bone resorption inhibitor, down-regulated the release of aged bone-EVs in VD3-treated aged mice to reduce the ovariectomy-induced VC [10]. Bisphosphonates (BiPs) are commonly used to treat bone loss and are also a strategy intended to prevent pathological calcification. Retrospective clinical data examining the effects of BiP therapy on cardiovascular calcification have revealed conflicting findings and intriguing paradoxes [308–310]. Ruiz et al. [311] established an AS model in APOE^-/-^mice and a 3D collagen hydrogel incubated with calcifying EVs to mimic an in vitro fibrous cap calcification model to determine whether BiP treatment altered microcalcification formation. In both models, it was shown that one of the mechanisms by which BiP modulates cardiovascular mineralization is to alter the kinetics of EVs-mediated microcalcification formation in a time-dependent manner, depending on whether BiP treatment was initiated before or after the expected onset of microcalcification formation [311]. PT and PT activated products can inhibit exosome-mediated VC by preventing nucleation site formation on the exosomal surface [235]. In addition to treating calcified EVs as a direct target for drugs [55], EVs may also be a promising strategy to treat VC by transferring bioactive substances or modulating signaling pathway. For example, BMSC-EVs are enriched in anti-calcification miRNAs such as miR-15a/15b/16 [188], miR-381-3p [189] and miR-146a [193], which can be transferred to VSMCs to improve VC. BMSC-EVs also improved VC by modulating the NONHSAT 084969.2/NF-κB axis [190] and the SIRT6-HMGB1 deacetylation pathway [191]. Curcumin may inhibit VC by increasing miR-92b-3p loading in VSMC-derived EVs and decreasing expression of its target KLF4 [245]. Melatonin attenuates osteogenic differentiation and senescence of VSMCs or calcified VSMCs by upregulating miR-204/miR-211 in EVs secreted by VSMCs [71]. AGEs inhibit diabetic medial calcification by stimulating HUVEC to secrete miR-126-5p-rich sEVs [70]. TC-EVs ameliorates aortic valve calcification via the miR-30b/Runx2/Wnt/β-catenin axis [272]. Furthermore, GFOGER peptide inhibits osteogenic transformation of VSMCs by modulating the content of proteins associated with osteogenic phenotype in VSMC-EVs, and also partially inhibits VC by preventing EVs-COL 1 interaction [242, 243].

There are other potential therapies for VC associated with EVs. The above sortilin protein can transport calcifying protein to EVs, and can also form homodimers containing intermolecular disulfide bonds to promote sortilin transport to EVs, thus forming EVs with high mineralization capacity. Anti-sortilin treatment significantly reduced VC due to EVs [69, 238, 239]. Modification of electrospun poly (ε-caprolactone) (PCL) vascular grafts with heparin to enhance their antithrombotic properties, whereas heparinization can caused severe graft calcification [312]. Human placenta-derived MSCs (hP-MSCs) derived sEVs were loaded onto heparin-functionalized vascular grafts (PCL-Hep) to form PCL-Hep/sEVs [312]. HP-MSC-sEVs significantly inhibited calcification and improved patency of vascular grafts by immunoregulation in hyperlipidemia rats, and more surprisingly, hP-MSCs-derived EVs have potent immunomodulatory effects by inducing a switch from a pro-inflammatory and AS-causing phenotype to an anti-inflammatory and anti-osteogenic phenotype in macrophages [312]. The preparation of immunomodulatory vascular grafts improves vascular performance and function by modulating EVs, which provides a therapeutic measure.

Elevated levels of fibroblast growth factor 23 (FGF23) and phosphate have been recognised as cardiotoxins causing left ventricular hypertrophy and cardiac fibrosis, among others, and their elevated levels are strongly associated with cardiovascular disease in CDK patients [313–315]. FGF23 can promote MSC of vascular progenitor cells towards osteoblastic differentiation and calcification, leading to excessive deposition of calcified ECM [316]. By testing uremic plasma from 19 end-stage renal disease dialysis patients, it was found that using the novel dialysis technology HCO membrane compared to conventional high-flux haemodialysis (HFL), HCO membrane can effectively remove pro-inflammatory factors including FGF23 to alter plasma composition and thereby protect vascular progenitor cells from calcification [316]. Another study showed that in vitro, conversion of hemodialysis patients from bicarbonate hemodialysis (BHD) to mixed online hemodiafiltration (mOL-HDF) treatment significantly reduced the expression of miR-223 in plasma-derived EVs, which was associated with osteogenic differentiation of VSMCs [259]. This technique may parallel the altered extracellular vesicle production in the anti-calcification mechanism, but further research is needed to determine whether the novel dialysis technique can actually reduce cardiovascular loss and contribute to angiogenesis and improve the prognosis of dialysis patients [316]. We summarize the potential benefits of EVs as therapeutic agents for calcification in Table 2.

**Table 2:** Potential benefits of extracellular vesicles as therapeutic drugs in calcification

Extracellular vesicles origin	Molecular mediators in EVs	Stimulus	Mechanisms	Effects	Refs
Aged bone-EVs	-	Alendronate	Alendronate down-regulated the release of EVs	Reduced the ovariectomy-induced VC	[10]
BMSC-EVs	-	-	EVs alter the miRNAs of VC-related mTOR, MAPK and Wnt signaling pathways in HA-VSMCs	Alleviated high Pi-induced VC	[187]
BMSC-EVs	miR-15a/15b/16	-	Transfer of miR-15a/15b/16 into VSMCs subsequently inhibited target NFATc3 and further downregulated OCN expression	Inhibited osteogenic transdifferentiation and calcification of VSMC	[188]
BMSC-EVs	miR-381-3p	-	Transfer of miR-381-3p into VSMCs downregulated NFAT5	Improved apoptosis and VC in CKD-VC	[189]
BMSC-EVs	miR-146a	AGEs-BSA	Transfer of high levels of miR-146a into VSMCs downregulated TXNIP expression	Inhibited AGEs-BSA-mediated VC	[193]
BMSC-EVs	-	-	Transfer of EVs into VSMCs regulated the NONHSAT 084969.2/NF-κB axis	Inhibited pi-induced VSMC transdifferentiation and calcification	[190]
BMSC-EVs	-	-	Transfer of EVs into VSMCs regulated the SIRT6-HMGB1 deacetylation pathway	Inhibited high Pi-induced aortic calcification	[191]
VSMC-exosomes	-	PT and PT activation products	Inhibited of nucleation sites on the surface of EVs	Prevented exosome-mediated VC	[235]
VSMC-MVs	-	Anti-sortilin	Anti-sortilin treatment inhibited the formation of hypermineralizable MVs	Prevented Nε-Carboxymethyl-lysine (CML)-induced diabetic atherosclerotic calcification	[69]
VSMC-EVs	Osteogenic phenotype-related proteins	GFOGER peptide	GFOGER peptide regulated the content of proteins associated with osteogenic phenotype in EVs and prevented EVs-col 1 interaction	Inhibited osteogenic transformation of VSMCs	[242, 243]
VSMC- EVs	miR-92b-3p	Curcumin	Curcumin increased miR-92b-3p loading in EVs and decreased expression of its target KLF4	Inhibited vitamin D3-induced VC	[245]
VSMCs/CVSMCs-EVs	miR-204/ miR-211	Melatonin	Melatonin upregulated miR-204/ miR-211 in EVs	Reduced the osteogenic differentiation and senescence of VSMCs/CVSMCs	[71]
VSMC-EVs	-	Verapamil	Verapamil inhibited ALP activity of calcified EVs and reduced the ability of EVs to subsequently calcify on COL 1	Prevented ECM mineralization	[306]
VSMC-EVs	-	Bisphosphonates	Maybe the pyrophosphate-like activity of BiP	BiP blocked EV aggregation and altered existing mineral growth	[311]
VSMC-EVs	MGP and Annexin A2	BGP-15	BGP-15 increased MGP content and decreased Annexin A2 content in EVs and prevented calcium deposition in the extracellular matrix	Inhibited Pi-induced osteochondrogenic phenotypic transition and mineralization in VSMCs under either NG or HG	[307]
HUVEC-EVs	miR-126-5p	AGEs	AGEs stimulated the secretion of mir-126-5p-rich EVs	Inhibited diabetic medial calcification	[70]
Telocytes-EVs	-	-	EVs promoted miR-30b expression and inhibited Runx2 expression and the Wnt/β-catenin pathway after uptake by calcified valve tissues and cells	Improved aortic valve calcification	[272]
Human placenta-derived MSCs-sEVs	VEGF, miR-126 and miR-145	-	EVs induced macrophages to shift from a M1(AS-causing phenotype) and to a M2(anti-osteogenic phenotype) and are enriched for VEGF, miR-126 and miR-145	Inhibited thrombosis and calcification to enhance the patency of vascular grafts and increased regeneration of endothelium and smooth muscle	[312]
Plasma-EVs	miR-223	mOL-HDF	mOL-HDF treatment reduced the expression of miR-223 in EVs	Inhibited osteogenic differentiation of VSMCs	[259]

The above demonstrates that EVs are an attractive cell-free therapeutic product that shows positive effects on VC. It also illustrates the excellent clinical potential of EVs, particularly in terms of different cell sources and their application in next-generation diagnostic and therapeutic platforms. EVs not only play a role in the treatment of diseases by themselves, but also show a broad application prospect as drug carriers based on their advantages. EVs have the following objective advantages: Firstly, safety: they are naturally occurring secretory membrane vesicles with lower toxicity and lower immunogenicity, which can avoid immune rejection and greatly improve safety [317, 318]; Secondly, EVs can cross biological barriers (e.g. blood–brain barrier) [319]; Thirdly, stability: EVs can protect their contents from degradation and thus prevent degradation and failure of the active ingredient [40]; Finally, controllable: altering the cellular microenvironment can regulate the function of EVs [40]. The drug loading strategies for loading drugs into EVs can be divided into 1. exogenous loading systems is to load drugs directly into EVs: physical methods (such as adsorption, electroporation, liposome fusion, etc.), chemical methods (such as chemical coupling, etc.); 2. endogenous loading systems is to load drugs into EVs-secreting cells: cell co-culture, direct modification of parental cells, genetic engineering, etc. [320]. By these methods drugs are directly transferred into EVs or genes encoding proteins of interest are transferred into cells secreting EVs for better treatment of calcification. And the in vitro construction of engineered vesicles with specific expressed active substances to mimic the effects of EVs may also be future therapeutic tools to improve VC.

In addition, we list crosstalk-based preclinical studies on VC in Table 3, and these reflect the fact that the development of VC has been shown to involve multi-cells and multi-organs co-regulation in animal models of VC and that the novelty of EVs will drive the research of VC from single cells to multi-cells, multi-organs and even multi-systems. We list the current state of clinical trials of drugs and therapeutic and preventive approaches (e.g., IVL, parathyroidectomy, dialysis, etc.) related to VC in Table 4. Few clinical trials related to VC of EVs as therapeutic delivery systems have been carried out, which also reflects that EVs have great room for application in VC. Due to the use of allogeneic EVs, translating basic therapies into clinical practice remains challenging [321]. There is growing evidence that EVs do play a role in disease progression, and we still require strong research efforts to make clinical practice possible in the future.

**Table 3:** Preclinical experiment of vascular calcification based on “crosstalk”

Model	Intervene	Mechanism	Results	Refs
Intramuscular injection of vit D3 and nicotine by gavage	Oral/Rectal propionate administration or FMT intervention or intragastric administration Akkermansia	Propionate can reduce VC by mediating intestinal microbiota remodelling and supplementation with Akkermansia can also alleviate VC	Improved VDN-induced VC	[322]
Intraperitoneally injected with VD3	Intravenous injection AB-EVs or YB-EVs	AB-EVs are transported into circulation and deposited in blood vessels to stimulate RUNX2 expression and osteogenic transition of VSMCs via transferring miR-2861	AB-EVs aggravated VC	[10]
Subcutaneous injection of Vit D	Intraperitoneally injection of Torin- 1 (an mTOR inhibitor)	Torin-1 significantly reduced the co-localization of mTOR vs Lamp-1 and increased lysosome-MVB interaction	Reduced Pi-induced mineral deposition	[230]
Intraperitoneal injection of Vit D3	Curcumin by gavage	Curcumin increased miR-92b-3p loading in EVs and decreased expression of its target KLF4	Improved vit D-induced VC	[245]
High-fat diet (HFD) and intraperitoneal injection of STZ and vit D	Tail vein injection of A-EC/sEVs	AGEs stimulate the secretion of miR126-5prich sEVs, targeting BMPR1 to block the smad1/5/9 signaling pathway	Improved T2D-mediated VC	[70]
HFD and intraperitoneal injection of STZ	BMCs of LV galectin-3 were transplanted into ApoE^−/−^ mice	Galectin-3 in macrophages promoted migration of VSMC- derived EVs to the intima	Induced diabetic intima calcification	[293]
5/6 nephrectomy + high phosphate diet	•GW4869 •miR-670-3p endothelial cell-specific knock-in (miR-670-3p^EC−KI^) and knock-out (miR-670-3p^EC−KO^) mice	•GW4869 eliminated the formation and release of EVs •miR-670-3p regulated the downstream target IGF-1	•GW4869 improved high phosphorus-induced VC •miR-670-3p in ECs-derived EVs promoted VC	[68]
5/6 nephrectomy + high phosphate diet	Tail vein injection of BMSC-Exo	BMSC-Exo down-regulated NFAT5 by delivering miR-381-3p	Improved CKD-VC	[189]
5/6 nephrectomy + high phosphate diet	Tail vein injection of BMSC-Exo	BMSC-Exo regulated of the NONHSAT 084969.2/NF- κB axis	Inhibited high phosphorus-induced VC and improved renal function	[191]
5/6 nephrectomy + high phosphate diet	Tail vein injection of adenovirus encoding omentin1/ omentin-1-deficient (omentin-1^−/−^) mice	Omentin-1 activated AMPK and Akt signaling pathways	Adipose tissue-derived omentin-1 reduced arterial calcification	[217]
5/6 nephrectomy + high phosphate diet	Tail vein injection of VSMCs-Exo for melatonin treatment	Melatonin up-regulated miR-204/miR-211 in EVs	Improved VC and aging	[71]
CKD (high phosphorus diet)	Increased BßGlucans diet (CKD + BßGlucans) from barley flour	Barley-ß-glucans inhibited SMPD3 expression	Reduced inflammation, kidney damage and aortic calcification	[229]
High-cholesterol (HC) diet	Tail vein injection of miR-214 specific-siRNA	miR-214-specific-siRNA leads to increased expression of the downstream target TWIST1	Improved HC diet-induced aortic valve calcification (AVC)	[291]

**Table 4:** **|** Summary of VC-related Clinical Trials

**Author(s) and year study start to completion (or expectation)**	**Number of cases**	**Clinical trial status**	**Research conten**t	**Clinical trial number**
Ercan Ok 2006–2008	446	Completed	Effects of atorvastatin therapy on coronary artery calcification progression, carotid intima-media thickness progression, endothelial function, and inflammation in hemodialysis patients	NCT00481364
Amgen 2006–2009	360	Completed	Whether cinacalcet plus low-dose vit D attenuated the progression of VC over one year compared with the use of flexible vit D doses in patients with chronic kidney disease receiving hemodialysis	NCT00379899
Peter G Kerr 2007–2009	50	Completed	Effects of alendronate sodium on cardiovascular and bone mineral parameters	NCT00395382
Sinee Disthabanchong 2008–2009	50	Completed	The treatment effect of sodium thiosulfate on coronary calcification in patients on hemodialysis	NCT00720772
Karen E Yeates 2008–2013	25	Completed	Effect of bisphosphonates on VC in adult dialysis patients	NCT00687661
Sylvia E Rosas 2008–2013	44	Completed	Different forms of vit D may have differential effects in VC progression in CKD patients	NCT00752102
Angela YM Wang 2010–2017	67	Completed	Effects of oral cinacalcet and parathyroidectomy combined with forearm autografting on coronary artery and valve calcification progression and left ventricular mass index in end-stage renal disease patients receiving peritoneal dialysis for more than 12 months	NCT01447368
Shih-Li Su 2011–2012	60	Completed	Effect of vit K2 on human osteoporosis, VC and sclerosis	NCT01928134
Rogier Caluwé 2011–2012	165	Completed	Find the best dose of vit K2 for hemodialysis patients	NCT01675206
Rachel Holden 2012–2019	85	Completed	Whether vit K supplements three times a week reduce the progression of coronary artery calcification in dialysis patients over a 12-month period	NCT01528800
Alessandra Brofferio 2012–2021	7	Completed	Whether etidronate sodium is safe and effective in treating Arterial Calcifications due to Deficiency in CD73 (ACDC)	NCT01585402
Jürgen Floege 2013–2020	63	Completed	Taking vit K1 to slow progression of VC in HD patients	NCT01742273
Ana Paula S Gueiros 2014–2016	33	Completed	The effect of spironolactone in the progression of coronary calcification in patients undergoing peritoneal dialysis	NCT03314493
Iain O Bressendorff 2015–2021	148	Completed	Effect of oral magnesium supplements on VC in patients with CKD	NCT02542319
Rogier Caluwe 2015–2019	117	Completed	Whether the replacement of vit K antagonists with Rivaroxaban slows VC and whether the addition of vit K2 to Rivaroxaban further slows the progression of VC	NCT02610933
Mabel Aoun 2016–2016	50	Completed	Assess the rate of decrease of dp-ucMGP in a hemodialysis cohort after supplementation with vit K2 and the correlation between this rate of decrease and the Aortic Calcification Severity (AC24) score	NCT02876354
Rocío Pérez Abud 2016–2017	59	Completed	The effect of vit K2 on VC in patients on hemodialysis	NCT04539418
Alex Gold 2016–2019	274	Completed	Effect of 2 dose levels of SNF472 on progression of coronary calcium volume scores in patients with HDESRD over a 12-month period compared with placebo	NCT02966028
Krishna Singh 2016–2020	102	Completed	Treatment of long-term calcified femoropopliteal artery lesions with directional arteriectomy and drug balloon	NCT02850107
Sabrina Wong Peixin Haroon 2016–2022	178	Completed	Prevalence and progression of VC in maintenance hemodialysis patients in an Asian population and the efficacy of vit k2 supplementation in reducing the progression of VC in this group of patients	NCT02870829
Nadia Mohsen Abdu Ibrahim 2017–2021	40	Completed	Clinical, laboratory, and VC outcomes in patients undergoing surgical parathyroidectomy within 6 months	NCT03724188
Thananda Trakarnvanich 2017–2019	201	Completed	Effect of DPP4-inhibitors (gemigliptin) on biomarkers of kidney injury and vascular calcification in diabetic nephropathy	NCT04705506
Alshaimaa Sayed Hassan Mubarak 2018–2020	120	Completed	Effects of intravenous injection of vit K1 on serum dephosphorylation-non-carboxylated MGP (dp-ucMGP) levels and aortic calcification scores and aortic and mitral valve disease severity	NCT05060809
Daniel Zickler 2018–2019	50	Completed	The effect of MCO or highflux dialysis on VC	NCT03104166
Rehab Werida 2019–2020	60	Completed	The effect of omega-3 fatty acids on the VC biomarkers Fetuin-A and OPG in dialysis patients	NCT03982966
Sarah Fahmy 2020–2021	40	Completed	Effects of vit K2 (menadione, MK-7) supplementation with vit k1 on circulating levels of calcification regulators and to evaluate its safety in routine dialysis patients	NCT04477811
Takayuki Hamano 2014–2023	60	Enrolling by invitation	To compare the effects of lanthanum carbonate and calcium carbonate on the progression of coronary artery calcification and vascular endothelial dysfunction	NCT02237534
Ekaterina Parshina 2019–2023	63	Active, not recruiting	Changes of cardiovascular calcification in dialysis patients with end-stage renal disease after parathyroidectomy versus conservative control group	NCT03937349
- 2021–2023	20	Active, not recruiting	To evaluate the safety and efficacy of shockwave coronary lithotripsy (IVL) system in the treatment of new, calcified, and narrowed coronary artery disease prior to stent placement	NCT05434676
Liu Jianghua 2021–2030	1400	Recruiting	To observe the influence of statins on the occurrence and development of CAC in patients with T2DM and the dynamic changes of serum miR-32 in the progression of CAC in patients with T2DM, and the possibility of being used as a biomarker for the serological diagnosis or prognosis of CAC	NCT04889053
Charles O'Neill 2021–2023	62	Recruiting	Whether citrate hemodialysis can reduce the course of VC	NCT04956120
Konstantinos Stavroulakis 2022–2024	50	Recruiting	Evaluate the 12 months safety and efficacy of the combination of Shockwave Intravascular Lithotripsy and a polymer coated Drug Eluting Stent device, for PACSS 3 and PACSS 4 calcified femoropopliteal disease	NCT05291247
Flavio Ribichini 2023–2024	50	Not yet recruiting	To investigate the treatment of calcified small coronary vessels with IVL plus drug balloon	NCT05625997

## Conclusions and perspectives

Current understanding of the pathogenesis of VC includes calcium and phosphorus imbalance, VSMCs transdifferentiation, extracellular matrix, EVs, bone homeostasis imbalance, inflammation, epigenetics (DNA methylation and demethylation, histone modification, non-coding RNA), autophagy, oxidative stress, mitochondrial dysfunction, iron death and pyroptosis, etc. The main therapeutic agents for VC are statins, sodium thiosulfate, bisphosphonates, SNF472, phosphate binders, calcimimetics, Denosumab, and TNAP inhibitors, etc. And the main therapeutic means are "coronary artery spinning" and "Intravascular lithotripsy (IVL)". To date, there is no definitive and feasible treatment for VC due to its irreversible process and complex pathology, and these measures mentioned above may have an interventional effect on VC, but their clinical application needs to be further explored. We have recognised that VC involves multicellular and multiorgan co-regulation and that EVs are a crucial mediator in crosstalk. In recent years, EVs have received widespread attention as their unique functions and extensive cargos have gradually been discovered. EVs are secreted in a controlled manner, so that changes in the microenvironment of the original cells cause changes in the production and contents of EVs. In VC, EVs have a dual role as both "disease-causing" and "disease-curing" agents. In this review, we address the mechanism of multi-organ/cellular regulation of VC from a new perspective of EVs, and a deeper understanding of the mechanism of VC can help in the discovery of optimal drug targets and drug development. In addition, EVs have potential therapeutic roles for calcification due to their properties as carrier systems, however, the use of EVs as a nanodrug delivery system for the treatment of calcification is still in the preclinical research phase or lacks extensive clinical studies. It is because researchers still face many issues and challenges, limitations in the preparation, engineering, and analytical techniques of EVs pose technical barriers to clinical translation. No extracellular vesicle products have received regulatory approval and remain challenging, and their associated risk–benefit ratios remain controversial, necessitating robust research efforts and preclinical and clinical approaches to assess their safety and efficacy.

In conclusion, there is an imbalance between calcification inhibitors and promoters in calcifying EVs: some inhibitors such as fetuin-A, MGP and some miRNAs were decreased, while some promoters such as annexin, Ca^2+^, Pi, TNAP and some miRNAs were increased. The mechanisms for regulating EVs under microenvironmental changes are as follows: (1) Regulation of Calcified EVs Release (2) Regulation of the interaction between EVs with ECM (3) EVs load more Ca^2+^ (4) Regulation of the content of bioactive molecules in EVs (such as, proteins and miRNAs, etc.) More importantly, EVs play a crucial role in cell-to-cell /organ-to-organ crosstalk, which may amplify and accelerate the progression of vascular calcification. We conclude that the EVs, in turn, can adjust VC through the following mechanisms: (1) Promote extracellular mineral deposition; (2) Promote osteogenic differentiation and senescence of VSMC; (3) Transfer bioactive molecules between cells; (4) Regulate autophagy, oxidative stress, endoplasmic reticulum stress mitochondrial dysfunction, inflammation and MAPK/mTOR/Wnt signaling pathways, etc. However, what we have discovered is only the tip of the iceberg in this complex network and is far from sufficient. Through EVs as mediators, the crosstalk between other cells (such as ECs)-VSMCs in VC is also reflected in the effect of VSMCs on ECs, which can also amplify the effect of ECs on VSMCs through feedback loops. Not only that, EVs of different cellular origin regulate VSMC calcification in turn calcifying VSMCs can also feedback to regulate other neighbouring cells. Understanding that EVs of different cellular origins regulate cardiovascular calcification can lead to a better understanding of the complex mechanisms underlying VC development. RNA cargo in EVs is more extensively studied as proteomics technologies lag behind sequencing technologies. Proteins are similarly functionally important and more extensive analysis will be necessary at a later date. In vitro organ culture, co-culture, and 3d culture of multiple cell types are the most promising tools to overcome the limitations of traditional in vitro models, and their widespread use in the coming years may help us to identify potential targets, ameliorate and prevent calcification in EVs-mediated VC.

## Data Availability

Not applicable.
